# The hypothesis of neuronal interconnectivity as a function of brain size—a general organization principle of the human connectome

**DOI:** 10.3389/fnhum.2014.00915

**Published:** 2014-11-11

**Authors:** Jürgen Hänggi, Laszlo Fövenyi, Franziskus Liem, Martin Meyer, Lutz Jäncke

**Affiliations:** ^1^Division Neuropsychology, Department of Psychology, University of ZurichZurich, Switzerland; ^2^Research Unit for Neuroplasticity and Learning in the Healthy Aging Brain (HAB LAB), Department of Psychology, Institute of Psychology, University of ZurichZurich, Switzerland; ^3^Department of Psychology, International Normal Aging and Plasticity Imaging Center, University of ZurichZurich, Switzerland; ^4^Center for Integrative Human Physiology (ZIHP), University of ZurichZurich, Switzerland; ^5^University Research Priority Program, Dynamic of Healthy Aging, University of ZurichZurich, Switzerland; ^6^Department of Special Education, King Abdulaziz UniversityJeddah, Saudi Arabia

**Keywords:** interhemispheric vs. intrahemispheric connectivity, human brain connectome, corpus callosum, functional specialization, hemispheric lateralization, diffusion tensor imaging, cross-species comparison, elephant

## Abstract

Twenty years ago, Ringo and colleagues proposed that maintaining absolute connectivity in larger compared with smaller brains is computationally inefficient due to increased conduction delays in transcallosal information transfer and expensive with respect to the brain mass needed to establish these additional connections. Therefore, they postulated that larger brains are relatively stronger connected intrahemispherically and smaller brains interhemispherically, resulting in stronger functional lateralization in larger brains. We investigated neuronal interconnections in 138 large and small human brains using diffusion tensor imaging-based fiber tractography. We found a significant interaction between brain size and the type of connectivity. Structural intrahemispheric connectivity is stronger in larger brains, whereas interhemispheric connectivity is only marginally increased in larger compared with smaller brains. Although brain size and gender are confounded, this effect is gender-independent. Additionally, the ratio of interhemispheric to intrahemispheric connectivity correlates inversely with brain size. The hypothesis of neuronal interconnectivity as a function of brain size might account for shorter and more symmetrical interhemispheric transfer times in women and for empirical evidence that visual and auditory processing are stronger lateralized in men. The hypothesis additionally shows that differences in interhemispheric and intrahemispheric connectivity are driven by brain size and not by gender, a finding contradicting a recently published study. Our findings are also compatible with the idea that the more asymmetric a region is, the smaller the density of interhemispheric connections, but the larger the density of intrahemispheric connections. The hypothesis represents an organization principle of the human connectome that might be applied also to non-human animals as suggested by our cross-species comparison.

## Introduction

Whether variations in brain size, both across and within species, are associated with variations in white matter connectivity is still disputed intensively. The architecture of the brain's connections (a.k.a. connectome) might be differently organized in smaller compared with larger mammalian brains. The universal scaling law between gray (GM) and white matter (WM) of the cerebral cortex postulates a disproportionally faster increase in WM compared with GM with increasing brain size (Zhang and Sejnowski, 2000). With respect to GM volume, it has been shown that when controlling for the overall brain size smaller human brains showed proportionally more GM than larger brains, whereas WM volume was enhanced in larger compared with smaller human brains, but this difference did not reach statistical significance (Lüders et al., 2002). However, little is known about how the disproportional increase in WM that is implied by the universal scaling law is distributed within the cortex in larger compared with smaller brains.

The conduction delay of axons is mainly a function of its diameter and length (Waxman, 1977). Therefore, the conduction delay of long-distance fibers must increase with increasing brain size, unless there is a proportional increase in fiber diameter, which seems not to be the case (Aboitiz et al., 1992a,b; Schuz and Preissl, 1996; Schuz et al., 2006). The absence of axons with proportionally increased fiber diameters in larger mammalian (e.g., human) compared with smaller mammalian brains (e.g., mouse) led us to assume that brain connectivity must be differently implemented across species. However, the theoretical model underlying the cortical scaling across mammalian species (Zhang and Sejnowski, 2000) may not apply to cortical scaling within a species, e.g., within humans (Peters et al., 1998; Im et al., 2008). Therefore, in the context of the present study we aimed at revealing how the disproportionally increased WM is distributed within the cortex in larger compared with smaller human brains.

Twenty years ago, Ringo and colleagues postulated that maintaining absolute connectivity in larger compared with smaller brains would be computationally inefficient due to increased conduction delays and expensive with respect to the brain mass devoted to these connections (Ringo, 1991; Ringo et al., 1994). Based on empirical data from animal studies, Ringo and colleagues estimated the amount of additional WM mass needed to maintain absolute connectivity in larger compared with smaller brains. These estimates suggested that it is not possible to maintain absolute connectivity in larger compared with smaller brains due to space restrictions (Ringo, 1991) and this finding is in good accordance with other theoretical models of brain mass growth and connectivity (Anderson, 1999; Braitenberg, 2001). Please note that this fact does not necessarily imply that different species have the same quantitative distribution of connections.

Ringo and colleagues (Ringo et al., 1994) designed a brain model based on an artificial network constrained by well-known macaque anatomical connectivity data (Lamantia and Rakic, 1990a,b). This artificial self-organizing neural network (see Supplementary Figure 1) was able to optimize its activation pattern during a simulated pattern discrimination task (see Supplementary Introduction). Using this network with simulated large and small brains together with their associated transcallosal connectivities they trained the network with the pattern discrimination task. After removing the transcallosal connections from this model it turned out that the performance in the small brain model declined stronger and faster than in the large brain model suggesting that interhemispheric connectivity is more important in small compared to large brains (Ringo, 1991; Ringo et al., 1994) (Supplementary Figure 2). The authors concluded that in larger brains both hemispheres would work more independently from each other due to longer callosal transmission times than both hemispheres of smaller brains. Therefore, interhemispheric connectivity should be stronger in smaller brains while larger brains should show relatively increased intrahemispheric connectivity compared with smaller ones (Ringo, 1991; Ringo et al., 1994).

Allometric investigations have shown that the increase in GM volume outpaces the increase of the size of the corpus callosum, the most important interhemispheric commissure, in humans (Jäncke et al., 1997; Im et al., 2008) as well as in non-human primates (Rilling and Insel, 1999a,b). In agreement with these findings, Leonard and colleagues showed that human individuals with larger cerebral volumes tended to demonstrate relatively smaller CC mid-sagittal areas (Leonard et al., 2008), suggesting that callosal connectivity is not proportionally scaled up in larger compared with smaller brains.

Although there is strong, but indirect evidence for a relationship between brain size and the kind of neuronal interconnectivity as outlined above, direct evidence is still lacking in humans. In animals, however, the proportion of callosal fibers in relation to brain size or to the estimated number of cortical cells across different species decrease with increasing brain mass (Olivares et al., 2001), thereby reducing the degree of interhemispheric connectivity; a fact also shown mathematically (Herculano-Houzel et al., 2010). In the present study, we sought out (1) to verify the conjecture proposed by Ringo and colleagues 20 years ago (Ringo, 1991; Ringo et al., 1994) by directly investigating it in humans through the use of diffusion-weighted magnetic resonance imaging (MRI) combined with quantitative fiber tractography; (2) to show that the interaction between brain size and the type of connectivity is independent of gender, although gender and brain size are correlated and (3) to replicate earlier and indirect findings (Jäncke et al., 1997; Im et al., 2008; Leonard et al., 2008) using a more direct methodological approach to connectivity. We hypothesized a significant interaction between brain size and intrahemispheric vs. interhemispheric connectivity. More specifically we predicted an inverse relationship between brain size and the interhemispheric/intrahemispheric connectivity ratio independent of gender.

## Materials and methods

### Subjects

Sixty-nine female subjects with a mean age of 26.3 years (standard deviation, *SD* ± 7.0 years) and 69 male subjects (mean age ± *SD*, 24.8 ± 4.6 years) matched for age [*t*_(116.4)_ = 1.56, *p* = 0.12, *d* = 0.29] and handedness [Pearson's χ^2^_(1)_ = 0.30, *p* = 0.59, ϕ = 0.05] participated in the present study. Due to the fact that most of the participants were university students, years of education between genders are well matched. Handedness was evaluated according to the procedure proposed by Annett (1970). The participants had no history of neurological and psychiatric disorders or neuropsychological problems and denied taking illegal drugs or medication. These 138 participants were divided into two groups using a median split of the variable total brain volume (TBV) as a measure of brain size. The research reported in the present study was conducted according to the principles expressed in the Declaration of Helsinki. The local ethics committee of the canton Zurich approved the study and written informed consent was obtained from all participants prior to the study enrolment.

### Magnetic resonance imaging data acquisition

MRI scans were acquired on a 3.0 T Philips Achieva whole body scanner (Philips Medical Systems, Best, The Netherlands) equipped with a transmit-receive body coil and a commercial eight-element head coil array capable for sensitivity encoding (SENSE). One diffusion-weighted spin-echo, echo-planar imaging sequence was applied to all 138 participants. Slices were acquired in the transversal plane with a measured and reconstructed spatial resolution of 2.0 × 2.0 × 2.0 mm^3^ (matrix 112 × 112 pixels, 75 slices). Further imaging parameters were: Field of view FOV = 224 × 224 mm^2^, echo-time *TE* = 55 ms, repetition-time *TR* = 13,472 ms, flip-angle α = 90°, and SENSE factor *SF* = 2.1. Diffusion was measured in 32 non-collinear directions with a *b*-value of *b* = 1000 s/mm^2^ preceded by a non-diffusion-weighted (*b* = 0 s/mm^2^) volume (reference volume). Scan time was about 10 min. One volumetric 3D T1-weighted gradient echo sequence (fast field echo) scan was acquired in addition. Slices were acquired in the sagittal plane with a measured and reconstructed spatial resolution of 0.94 × 0.94 × 1.00 mm^3^ (matrix 256 × 256 pixels, 160 slices). Further imaging parameters were: Field of view FOV = 240 × 240 mm^2^, echo-time *TE* = 3.7 ms, repetition-time *TR* = 8.06 ms, flip-angle α = 8°, and SENSE factor *SF* = 2.1. Scan time was about 8 min.

### Estimation of total brain volume using freesurfer tools

The automated procedure for measuring TBV was performed with the volumetric and surface-based processing streams of the FreeSurfer software suite (version 5.0.0), which is documented and freely available for download online (http://surfer.nmr.mgh.harvard.edu/). The procedure automatically assigns a neuroanatomical label to each voxel in a T1-weighted MRI scan, a label that is based on probabilistic information automatically estimated from a manually labeled training set (Fischl et al., 2002). The technique has previously been shown to be comparable in accuracy to manual labeling (Fischl et al., 2002, 2004). TBV is simply the sum of the total cortical white matter volume and the total cortical and subcortical gray matter volume (including cerebellar gray and white matter volumes) as computed by FreeSurfer per default.

### Fiber tractography and reconstruction of the connectivity matrix

Preprocessing of the diffusion-weighted data was conducted with FSL (FMRIB Software Library; version 5.0.0; http://www.fmrib.ox.ac.uk/fsl/) (Smith et al., 2004) tools such as the FDT (FMRIB Diffusion Toolbox; version 3.0) (Behrens et al., 2003). For deterministic fiber tractography we used the Diffusion Toolkit (DTK; version 0.6.2.1; http://trackvis.org/) and TrackVis software (version 0.5.2.1; http://trackvis.org/) (Wang et al., 2007) and the connectivity matrix was computed in MATLAB (version 7.10.0; http://www.mathworks.com/index.html). The following steps were realized: (1) Eddy current and head movement correction were applied using the EDDY_CORRECT tool of FDT. (2) Diffusion gradients were adjusted for rotations introduced by the eddy current and head movement correction. (3) These preprocessed data were subjected to the DTK to compute the diffusion tensors and to construct the eigenvector and eigenvalue maps as well as a map of fractional anisotropy (FA). (4) Deterministic tractography was conducted in TrackVis using the “brute force” approach with an interpolated streamline tracking algorithm. Twenty streamlines (reconstructed fibers) per voxel were propagated and tracking was terminated if FA was lower than 0.10 or if the turning angle of a streamline between two consecutive voxels was larger than 45°, resulting in a whole-brain connectome comprised of about 2–3 Millions of streamlines in total (including subcortical pathways and connections to the cerebellum). (5) The individual FA map was registered onto the FMRIB58-FA template, which is in correspondence with the MNI152 standard space, using FSL's linear image registration tool (FLIRT) and the resulting transformations were stored. (6) These transformations were applied to the streamlines produced in step 4 in order to transform the streamlines into the MNI152 space. (7) The automated anatomical labeling (AAL) regions of interest (ROIs) (Tzourio-Mazoyer et al., 2002), which are already in MNI152 standard space, were used to count the number of streamlines between each pair of ROIs. The AAL template consists of 116 ROIs in total and we removed the 26 cerebellar ROIs, resulting in 90 ROIs (45 per hemisphere) covering the entire neocortex (78 cortical ROIs) as well as the subcortical structures amygdala, hippocampus, thalamus, caudate, putamen, and pallidum (12 subcortical ROIs). (8) Streamlines connected to the cerebellum, those running through the brainstem, and streamlines shorter than 5 mm in length were removed (denoted streamlines omitted). Streamlines that make connections within a ROI were deleted (denoted selfloops). The number of the remaining streamlines between any pair of ROIs (denoted streamlines used to populate matrix) was counted using MATLAB scripts (Zalesky et al., 2010). (9) This procedure resulted in an undirected, weighted 90 × 90 connectivity matrix for each subject. (10) In the last step, we summed up the number of streamlines devoted to interhemispheric and those devoted to intrahemispheric connections for each subject.

Neuronal (inter)-connectivity in the present study is conceptualized as the total number of reconstructed streamlines between brain regions. The intrahemispheric connectivity measure represents all connections within one hemisphere summed up across both hemispheres, whereas the interhemispheric connectivity measure is mainly expressed by the connections of the corpus callosum, hippocampal commissure, and anterior commissure, but may also include parts of the posterior commissure and the habenular commissure.

**Figure 1:**
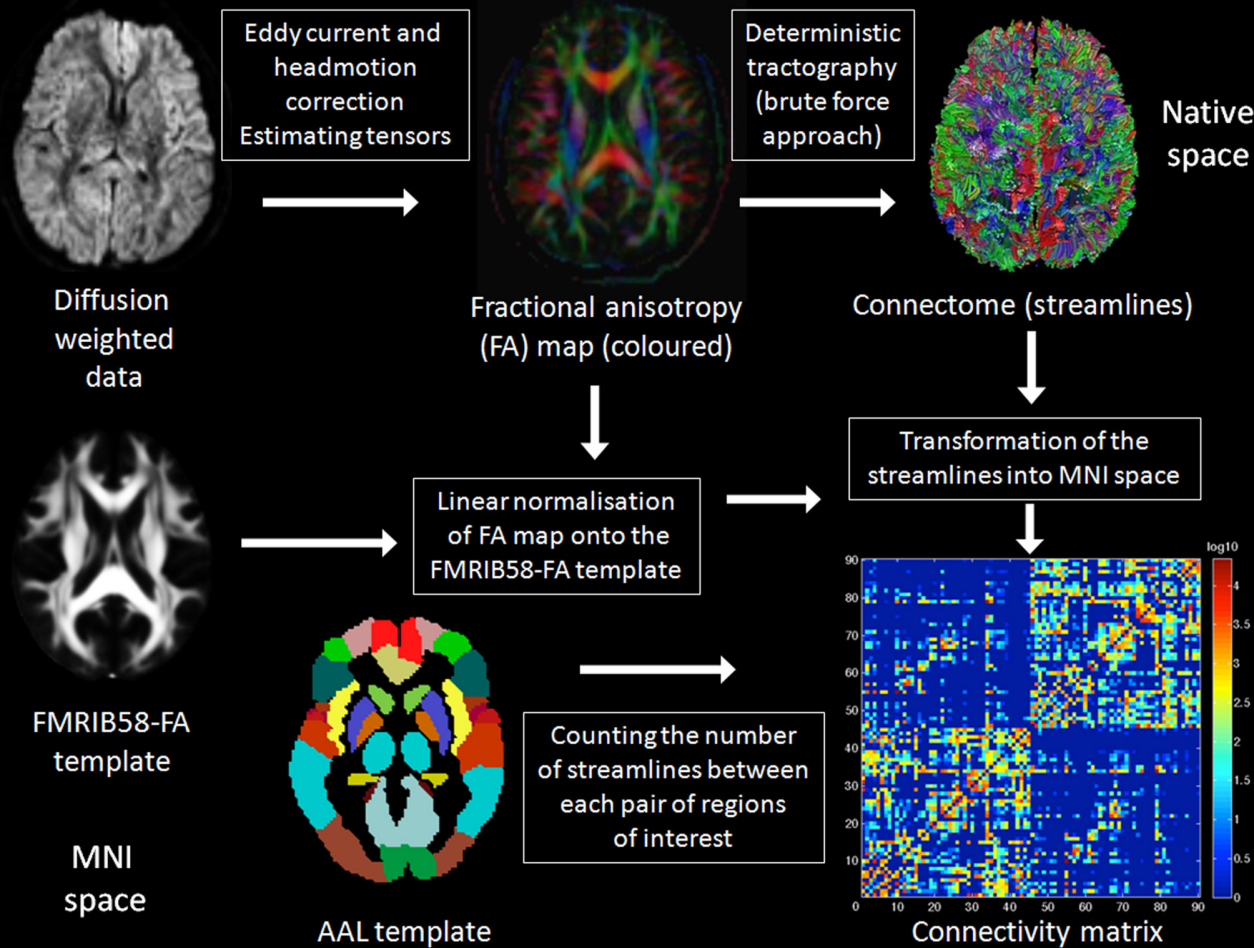
**Summary of the methods**. A detailed description of the methods applied in the present study can be found in the Materials and Methods Section below. In brief, raw DTI data were eddy current and head motion corrected and the diffusion tensors were estimated. Deterministic tractography was applied using the brute force approach resulting in the reconstruction of all fibers of the brain. The fractional anisotropy (FA) map was spatially normalized onto the FMRIB58-FA template to get the transformations from native to stereotactic (Montreal neurological institute, MNI) space, which were subsequently applied to the reconstructed fibers. The automated anatomical labeling (AAL) regions of interest (ROI), a parcellation of the brain based on anatomical landmarks, were used to count separately the number of fibers devoted to interhemispheric and those devoted to intrahemispheric connections.

In order to show that the results are independent from the number of the ROIs used to construct the connectivity matrix (Zalesky et al., 2010), the 90 AAL ROIs were further divided into 180 smaller ROIs (using a MATLAB script downloaded from http://people.eng.unimelb.edu.au/azalesky/compact_parcellate.zip), and an undirected, weighted 180 × 180 connectivity matrix for each subject was constructed and the number of streamlines devoted to interhemispheric and those devoted to intrahemispheric connections were counted separately. The methods applied in order to generate the connectivity matrices in the present study are summarized in Figure 1.

### Statistical analyses

Statistical analyses were performed with IBM SPSS Statistics software (version 20.0; http://www-01.ibm.com/software/analytics/spss/). In a first step, we used analysis of variance (ANOVA) models with brain size as between-subject factor (69 small vs. 69 large brains) and the type of connectivity as within-subject factor (interhemispheric vs. intrahemispheric connectivity). In a second analysis, we divided the male group (*n* = 69) into two groups of men by applying a median split of the TBV (35 small male vs. 34 large male brains). In a third analysis, we divided the female group (*n* = 69) in the same manner as the male group (35 small female vs. 34 large female brains). The interaction between brain size and connectivity is the contrast of interest and *post-hoc* independent sample *t*-tests were applied to evaluate the direction of the interaction effects in more detail. To further characterize and visualize the relationship between brain size and the types of connectivity, we correlated brain size with the interhemispheric and intrahemispheric connectivity measure using Pearson's correlation. Effect sizes were reported in addition: η*_p_*^2^ (partial eta square) for ANOVA, Cohen's d for *t*-tests, and ϕ (Phi coefficient) for Pearson's χ^2^-tests. If not explicitly stated two-tailed error probabilities were employed.

We also calculated the connectivity ratio based on the interhemispheric connectivity measure divided by the intrahemispheric connectivity measure. This connectivity ratio was then correlated with TBV using Pearson's correlation in the whole sample (*N* = 138), and in the male (*n* = 69) and female subsample (*n* = 69). We also compared these ratios between the small and large brain groups in the gender-pooled sample as well as in the gender-specific subsamples using independent sample *t*-tests.

In addition, we analyzed specific interhemispheric and intrahemispheric connections to answer further questions. One may hypothesize that the apparent lack of functional lateralization in the olfactory cortex or hippocampus would be associated with no changes in interhemispheric connectivity in the anterior and hippocampal commissure as a function of brain size. For this purpose, we counted the number of interhemispheric streamlines between the left and right olfactory cortex (fibers of the anterior commissure) and those between the left and right hippocampus (hippocampal commissure). Here, we predicted a negative finding due to the lack of lateralization of these systems. The olfactory and hippocampal measure was analyzed using independent sample *t*-tests without correction for multiple comparisons.

### Cross-species comparison

The relevance of this cross-species comparison is motivated by the predictions that can be derived from the hypothesis of neuronal interconnectivity as a function of brain size. The hypothesis predicts an under-proportional increase in interhemispheric compared with intrahemispheric connectivity with increasing brain size. The hypothesis of neuronal interconnectivity as a function of brain size has been proposed to be a basic principle of organization in biology and therefore this connectivity pattern should also be evident across species. The literature-based (Anthony, 1938; Bauchot and Stephan, 1961; Saban et al., 1990; Jäncke et al., 1997; Rilling and Insel, 1999a; Hakeem et al., 2005; Shoshani et al., 2006; Leonard et al., 2008; Fears et al., 2009; Manger et al., 2010) cross-species comparison of the ratio between corpus callosum mid-sagittal area and brain size includes the following five categories: Elephants (*n* = 6), humans (*n* = 464, averaged into seven mean values including mean values of the subjects investigated in the present study), carnivores (*n* = 10), other primates (*n* = 17), and rodents (*n* = 4). In the categories carnivores, other primates, and rodents, only one animal per species was investigated. For the cross-species comparison, the corpus callosum mid-sagittal area of the animals was divided by their total brain size and this ratio showed small values ranging from 0.00165 to 0.0186. For more convenient reading, this ratio was multiplied by 100 to transform the numbers into the range of 0.165–1.860. To evaluate cross-species differences in this ratio, we employed a non-parametric Kruskal–Wallis test across the five categories and subsequent Mann–Whitney *U*-tests for comparing humans with elephants, carnivores, and other primates separately.

### Validation analyses

To rule out the possibility that our DTI-based connectivity findings might be affected by two well-known (streamline) tractography biases, the sampling-related bias and the distance-related bias (Li et al., 2012), we applied two different validation analyses using the same 138 experimental subjects as analyzed and reported in our main analyses. The first validation analysis is based on white matter volumes derived from T1-weighted MRI scans, which are not affected by the two biases at all. The second validation analysis is based on the DTI data as well, but we used another tractography algorithm that is less prone to the distance-related tractography bias.

Using FreeSurfer's automated segmentation procedure (Fischl et al., 2002) the volume of the total cortical white matter and that of the corpus callosum were computed. Callosal volume based on a mid-sagittal slab of 5 mm width and thus did not contain all callosal fibers. Interhemispheric connectivity is operationalized here as the callosal volume and intrahemispheric connectivity as the total white matter volume minus the callosal white matter volume. Although the white matter volumes used for this volume-based validation analysis are only indirect measures of connectivity, these volumes are strongly associated with the connectivity measures derived from the tractography data. Callosal volumes derived from T1-weighted MRI data correlated moderately with the number of interhemispheric streamlines derived from the DTI data (*r* = 0.545, *p* = 4.8E-12, Pearson's correlation) and intrahemspheric white matter volume derived from T1-weighted MRI data correlated strongly with the number of intrahemispheric streamlines derived from the DTI data (*r* = 0.806, *p* = 8.5E-33). In addition, callosal volume is heavily related to mid-sagittal corpus callosum area, a widely used quantitative measure of the corpus callosum (Jäncke et al., 1997; Leonard et al., 2008) that we also applied in our cross-species comparison (see above).

It is important to note that the distance-related bias is mainly an issue when using probabilistic tractography, because it propagates uncertainty across space and therefore, the longer a fiber bundle is the less certain we are about its location. This bias can be substantially reduced by seeding from the entire brain instead of seeding from the endpoints of the pathways (global vs. local tractography). However, deterministic tractography algorithms ignore uncertainty completely and therefore the distance-related bias of deterministic tractography algorithm is solely due to the propagation of the integration errors, which can be heavily reduced by using tractography algorithms with more accurate integration schemes such as the Runge-Kutta integration. Therefore, we re-computed the number of interhemispheric and intrahemispheric streamlines using a deterministic tractography algorithm that used a second order Runge-Kutta integration scheme as implemented in TrackVis (http://trackvis.org/).

## Results

### Demography

Demography, global brain measures, and connectomic characteristics of the gender-pooled sample (*N* = 138) are summarized in Table 1. We applied a median split to the TBV in order to build up a small (55 women and 14 men; mean TBV ± *SD*, 1089 ± 64 cm^3^) and a large brain group (55 men and 14 women; mean TBV ± *SD*, 1264 ± 70 cm^3^), which significantly differed in their TBV [*t*_(136)_ = −15.3, *p* = 2.3E-31, *d* = 2.6], but also in gender [Pearson's χ^2^_(1)_ = 48.7, *p* = 2.9E-12, ϕ = 0.59]. Handedness was not significantly different between the small and large brain group [Pearson's χ^2^_(1)_ = 1.62, *p* = 0.20, ϕ = 0.11]. The small brain group was significantly older (mean ± *SD*, 26.8 ± 7.1 years) than the large brain group [mean ± *SD*, 24.3 ± 3.4 years, *t*_(113.2)_ = 2.42, *p* = 0.02, *d* = 0.46]. However, the effect size of this age difference is rather small to moderate and not critical since it is known that the anatomical differences are small or even not existent in the second or third decade of life (Ziegler et al., 2012). Nevertheless, we also run statistical analyses that corrected for age (see below). Due to the fact that most of the participants investigated here were university students their years of education are closely matched. As expected, the small (*n* = 69) and large brain group (*n* = 69) significantly differed with respect to both of the two different tissue volumes that build up TBV, i.e., total GM volume as well as total WM volume (see Table 1).

**Table 1:** **Demography, global brain measures, and connectomic characteristics of the small and large brain group in the gender-pooled sample (*N* = 138)**.

**Demographic and global brain measures**	**Small brains (*n* = 69)**				**Error probability**	**Large brains (*n* = 69)**			
	**Mean**	***SD***	**Minimum**	**Maximum**	***p*-value**	**Mean**	***SD***	**Minimum**	**Maximum**
Age (years)	26.8	7.05	19.0	57.0	0.02	24.3	4.35	17.4	42.8
Gender (female/male)	55/14	–	–	–	<0.001	14/55	–	–	–
Handedness (left/right)	19/50	–	–	–	0.20	26/43	–	–	–
Total brain volume (ccm)	1088.9	63.9	858.7	1174.8	<0.001	1263.6	70.0	1175.9	1446.5
Total gray matter volume (ccm)	638.1	37.2	503.6	701.8	<0.001	733.3	39.2	650.9	812.6
Total white matter volume (ccm)	450.8	35.3	355.1	527.2	<0.001	530.3	38.7	451.9	645.9
**CONNECTIVITY MEASURES (90 NODES)**									
Total number of streamlines	2,397,895	246,429	1,824,454	3,003,328	<0.001	2,778,401	250,490	2,303,657	3,451,223
Streamlines omitted	1,353,562	130,735	1,034,426	1,685,013	<0.001	1,535,205	128,041	1,251,601	1,862,649
Streamlines used to populate matrix	1,044,332	130,320	790,028	1,425,252	<0.001	1,243,196	138,335	1,024,760	1,598,552
Selfloops	652,327	94,887	482,696	918,108	<0.001	790,568	106,773	613,408	1,062,856
Interhemispheric streamlines	128,866	27,200	74,355	192,938	0.007	141,682	28,215	71,994	207,746
Intrahemispheric streamlines (left and right)	589,303	69,162	460,073	787,741	<0.001	706,230	73,570	576,291	883,385
Connectivity ratio	0.218	0.037	0.132	0.316	0.005	0.201	0.035	0.114	0.260

*Connectivity ratio was calculated by dividing the interhemispheric by the intrahemispheric connectivity measure. Differences in gender and handedness were determined by χ*^2^*-tests and all other differences were determined by t-tests for independent samples with 136 degrees of freedom. n, number of subjects; SD, standard deviation*.

The results derived from the two different connectivity matrices constructed (90 vs. 180 ROIs, see above) are qualitatively and quantitatively very similar to each other so that we only report the results derived from the common 90 AAL ROIs in the main manuscript. The results derived from the 180 nodes connectivity matrices are reported in the Supplementary Results section online and are presented in Supplementary Figures 3–5 and Supplementary Tables 3–5.

### Interaction between interhemispheric and intrahemispheric connectivity

**Figure 2:**
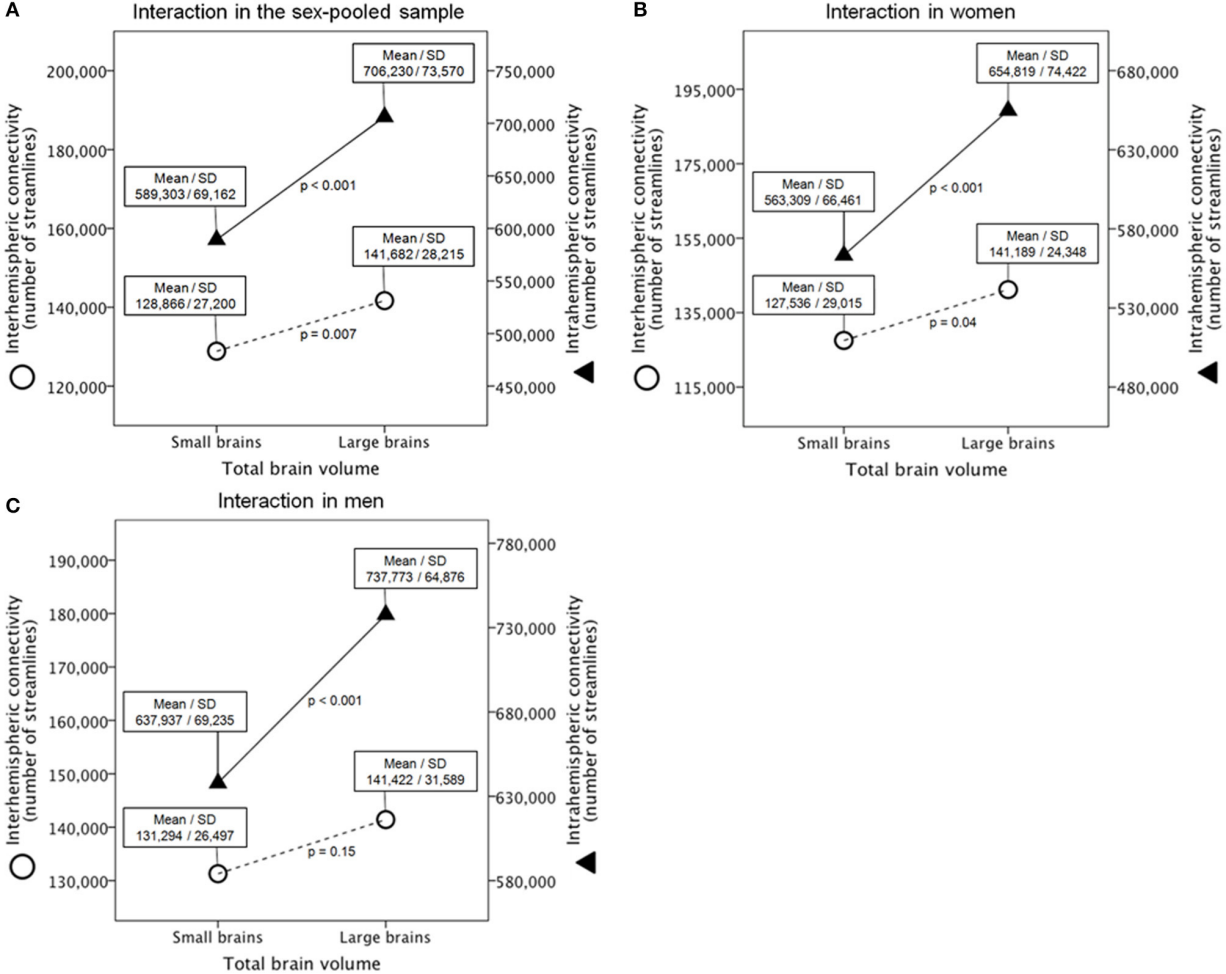
**Interaction between brain size and connectivity derived from the 90 ROIs connectivity matrix in the gender-pooled sample (A), in the female subsample (B), and in the male subsample (C)**. Shown are analysis of variance models with large vs. small brains as between-subject factor and interhemispheric vs. intrahemispheric connectivity as within-subject factor. Y-axes represent the number of reconstructed fibers.

First, a mixed analysis of variance (ANOVA) model (*N* = 138) revealed a highly significant interaction between brain size (between-subject factor; small vs. large brains) and connectivity (within-subject factor; interhemispheric vs. intrahemispheric connectivity) [*F*_(1, 136)_ = 101.9, *p* = 3.1E-18, η*_p_*^2^ = 0.43] (Figure 2A, c.f. Supplementary Figure 3A for the 180 nodes analysis). Subsequent *post-hoc t*-tests revealed highly significant and massively increased intrahemispheric connectivity in larger compared with smaller brains [*t*_(136)_ = −9.6, *p* = 5.0E-17, *d* = 1.65], whereas interhemispheric connectivity was also increased in larger compared with smaller brains, but with a moderate effect size [*t*_(136)_ = −2.7, *p* = 0.007, *d* = 0.47].

We additionally applied an ANCOVA model that corrects for age because the two groups differed slightly in age (26.8 vs. 24.3 years in the small and large brain group, respectively). This analysis replicated the finding reported above [*F*_(1, 136)_ = 104.3, *p* = 1.7E-18, η*_p_*^2^ = 0.44]. When using ANCOVAs that correct for age instead of *post-hoc t*-tests (see above) intrahemispheric connectivity is still significantly and massively increased in larger compared with smaller brains [*F*_(1, 135)_ = 94.3, *p* = 3.1E-17, η*_p_*^2^ = 0.41], whereas interhemispheric connectivity was increased in larger compared with smaller brains, but with a small effect size [*F*_(1, 135)_ = 7.3, *p* = 0.008, η*_p_*^2^ = 0.05].

Gender and TBV were not independent [*t*_(131.4)_ = −8.7, *p* = 1.1E-14, *d* = 1.52], therefore we applied a median split of the TBV within genders to rule out that the observed interaction is a gender instead a real brain size effect (Supplementary Tables 1, 2). Within these gender-specific samples, there were no significant differences with respect to age and handedness. As expected and intended by the median split, the small (*n* = 35) and large (*n* = 34) female brain group (Supplementary Table 1) as well as the small (*n* = 35) and large male (*n* = 34) brain group (Supplementary Table 2) significantly differed with respect to TBV, total GM volume and total WM volume.

Second, a mixed ANOVA model (*n* = 69) revealed also a significant interaction between brain size in women and connectivity [*F*_(1, 67)_ = 31.3, *p* = 4.5E-07, η*_p_*^2^ = 0.32] (Figure 2B, c.f. Supplementary Figure 3B for the 180 nodes analysis). Subsequent *post-hoc t*-tests revealed a significantly and massively increased intrahemispheric connectivity in large compared with small female brains [*t*_(67)_ = −5.4, *p* = 9.8E-07, *d* = 1.32], whereas interhemispheric connectivity was also increased in large compared with small female brains, but with a moderate effect size [*t*_(67)_ = −2.1, *p* = 0.038, *d* = 0.52].

Third, a mixed ANOVA model (*n* = 69) revealed also a significant interaction between brain size in men and connectivity [*F*_(1, 67)_ = 45.8, *p* = 3.9E-09, η*_p_*^2^ = 0.41] (Figure 2C, c.f. Supplementary Figure 3C for the 180 nodes analysis). Subsequent *post-hoc t*-tests revealed a significantly and massively increased intrahemispheric connectivity in large compared with small male brains [*t*_(67)_ = −6.2, *p* = 4.4E-08, *d* = 1.51], whereas interhemispheric connectivity was not significantly different between large and small male brains [*t*_(67)_ = −1.4, *p* = 0.15, *d* = 0.35].

Last, we additionally explored whether this patter of connectivity can also be found in cross-gender subgroup comparisons. We expected that the effect becomes stronger when comparing small female with large male brains due to the increasing differences in brain size between these groups [*t*_(67)_ = −21.0, *p* = 3.5E-32, *d* = 5.13]. This contrast can be regarded as an extreme group comparison. The contrast between large female and small male brains can be regarded as a negative control condition because these two groups do not differ in brain size [*t*_(58.8)_ = 0.82, *p* = 0.42]. A mixed ANOVA model (*n* = 69) revealed a significant interaction between brain size and connectivity when comparing small female with large male brains [*F*_(1, 67)_ = 161.2, *p* = 1.7E-19, η*_p_*^2^ = 0.71], whereas this interaction did not reach statistical significance between large female and small male brains [*F*_(1, 67)_ = 0.23, *p* = 0.63]. Subsequent *post-hoc t*-tests revealed a significantly and massively increased intrahemispheric connectivity in large male compared with small female brains [*t*_(67)_ = 11.0, *p* = 1.0E-16, *d* = 2.70], whereas interhemispheric connectivity was only different on a trend level toward significance [*t*_(67)_ = 1.90, *p* = 0.061, *d* = 0.46]. As expected, neither intrahemispheric nor interhemispheric connectivity was statistically significantly different between large female and small male brains [*t*_(67)_ = −0.98, *p* = 0.33 and *t*_(67)_ = −1.61, *p* = 0.11, respectively].

### Correlation between brain size and type of connectivity

**Figure 3:**
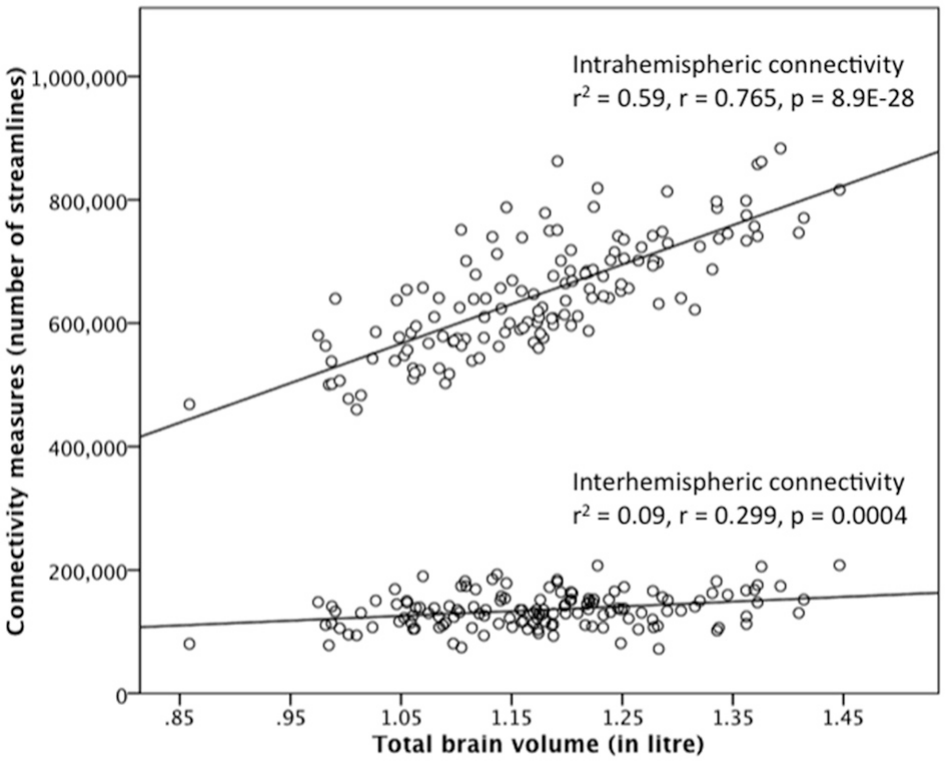
**Correlation between brain size and the interhemispheric and intrahemispheric connectivity measure derived from the 90 ROIs connectivity matrix in the gender-pooled sample (*N* = 138)**. Although brain size is positively correlated with both connectivity measures, the strengths of these two correlations are quite different. Brain size explains 59% of the variance in intrahemispheric connectivity, whereas brain size explains only 9% of the variance in interhemispheric connectivity.

In addition to these interactional analyses, we correlated brain size with the interhemispheric and intrahemispheric connectivity measure. Both correlations were positive and statistically significant, but differed in their strength. The relationship between brain size and intrahemispheric connectivity is strong (*r* = 0.765, *p* = 8.9E-28, Pearson's correlation) and brain size explains 59% of the variance in intrahemispheric connectivity. The relationship between brain size and interhemispheric connectivity is heavily reduced in strength (*r* = 0.299, *p* = 0.0004) and brain size only explains 9% of the variance in interhemispheric connectivity. The scatter-plots of these relationships are shown in Figure 3 for the 90 nodes connectivity matrix and in Supplementary Figure 4 for the 180 nodes connectivity matrix.

### Relationship between brain size and the connectivity ratio

Beside these mixed ANOVA models and the correlations reported above, we also computed the connectivity ratio based on the interhemispheric connectivity measure divided by the intrahemispheric connectivity measure and correlated this ratio with brain size. The descriptive statistics (mean, standard deviation, minimum, and maximum and the results of the statistical group comparisons of the connectivity ratio) are presented in Supplementary Tables 1–3. Across the whole sample (*N* = 138), the connectivity ratio was significantly negatively and linearly correlated with TBV (*r* = −0.258, *p* = 0.002, Pearson's correlation). Within the female (*n* = 69) and male sample (*n* = 69), however, the connectivity ratios were not statistically significantly correlated with TBV (*r* = −0.12, *p* = 0.33 and *r* = −0.084, *p* = 0.49, respectively).

**Figure 4:**
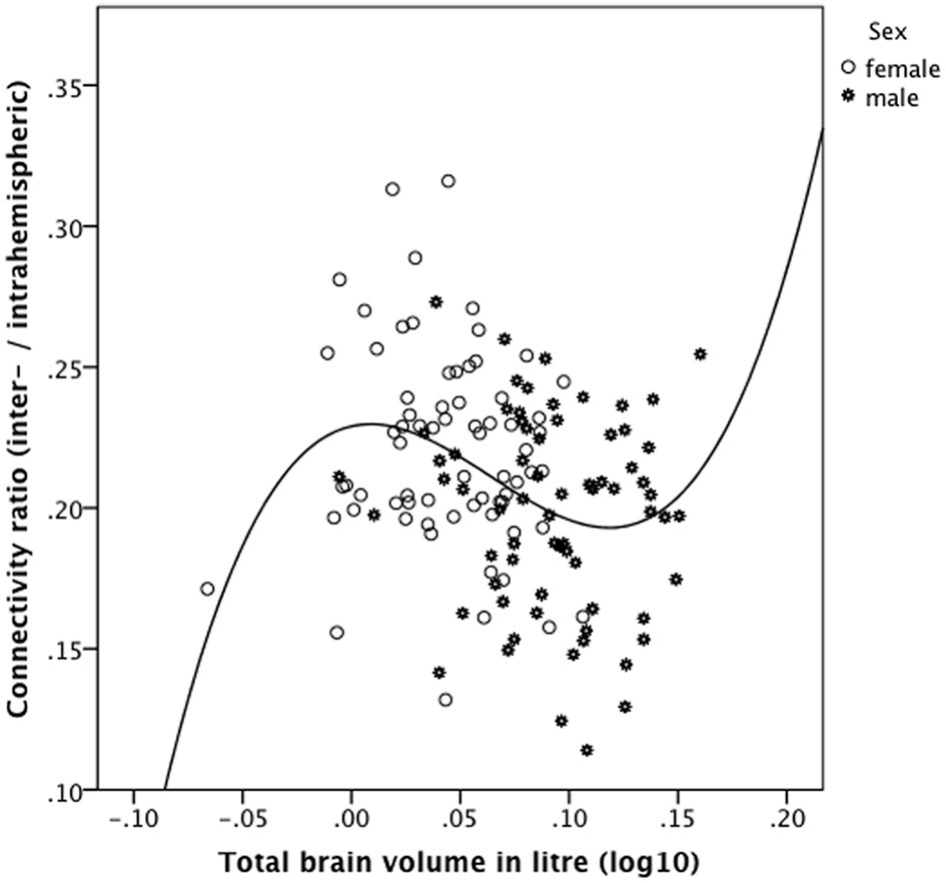
**Relationship between the connectivity ratio derived from the 90 ROIs connectivity matrix and brain size in the gender-pooled sample (*N* = 138) as revealed by a cubic function**. The connectivity ratio was computed based on the number of reconstructed interhemispheric connections (streamlines) divided by the number of intrahemispheric connections. Brains of intermediate size showed the predicted negative association of these measures. However, the smallest (mainly female) and the largest (all male) brains do not follow our predictions. These subjects showed a positive instead a negative association between the connectivity ratio and TBV.

Plotting the connectivity ratio against TBV (Figure 4), it is obvious that a cubic function better (*R*^2^ = 0.127, *p* = 0.0004) explains their relationship than a linear function (*R*^2^ = 0.067, *p* = 0.003), a difference in explained variance that is not attributable to an over fitting problem. For this purpose, we computed Akaike's information criterion (AIC) using the following formula AIC = n^*^ log (RSS/n) + 2^*^ k implemented in the R software (http://www.r-project.org/). RSS means residual sum of squares, n is the number of observations, and k is the number of model parameters. This analysis revealed that even when correcting for the number of parameters of the models the cubic fit is still better than the linear one (AIC = −914.8 for the linear fit and AIC = −920.4 for the cubic fit). Note that without the logarithmical transformation of the TBV values (log_10_ of TBV in liters), the cubic model could not be fitted due to near-collinearity among model terms.

As evidenced in Figure 4, the smallest and the largest brains investigated in the present study showed a positive association between the connectivity ratio and TBV, whereas brains of intermediate size showed the predicted negative association of these measures. We then computed the distribution points of the cubic function (y = 0.175211 ^*^ x −10.672421 ^*^ x^2^ + 55.933478 ^*^ x^3^ + 0.229008) and excluded all subjects below the saddle (log_10_ of TBV in liter = 0.0088 corresponding to 1.021 liter TBV) and above the valley (log_10_ of TBV in liter = 0.1184 corresponding to 1.313 liter TBV) and recomputed the linear correlation of the remaining subjects. In this way, the 12 smallest brains (10 women) and the 18 largest brains (all men) were excluded.

Across this reduced gender-pooled sample (*n* = 108) the connectivity ratio was negatively correlated with TBV (*r* = −0.391, *p* = 0.00001 one-tailed, Pearson's correlation). Within the reduced female subsample (*n* = 59) the connectivity ratio was negatively correlated with TBV (*r* = −0.312, *p* = 0.008 one-tailed), whereas within the reduced male subsample (*n* = 49) the connectivity ratio was also correlated negatively with TBV (*r* = −0.232), but only on a trend level toward statistical significance (*p* = 0.054 one-tailed).

To sum up, brains of intermediate size (108 out of 138) showed the predicted negative association of these measures. However, the smallest (mainly female) and the largest (all male) brains do not follow our predictions. These subjects showed a positive instead a negative association between the connectivity ratio and TBV.

### Investigation of specific interhemispheric connections

As a negative control condition, we analyzed interhemispheric connectivity within two systems that seem not to be lateralized in humans: the olfactory and hippocampal system. For this purpose, we counted the number of reconstructed fibers (streamlines) between both hippocampi and those between both olfactory cortices. In the gender-pooled sample, neither the number of anterior commissural streamlines was significantly different between the large and small brain group [mean ± *SD*: 122.2 ± 133.5 in small brains and 138.8 ± 130.0 in large brains; *t*_(136)_ = −0.74, *p* = 0.46, *d* = 0.13] nor the number of hippocampal streamlines [mean ± *SD*: 37.7 ± 61.1 in small brains and 35.2 ± 71.6 in large brains; *t*_(136)_ = 0.22, *p* = 0.83, *d* = 0.04]. There was also a negative finding here when using relative measures, i.e. when the number of hippocampal and anterior commissural streamlines was each divided by the total number of interhemispheric streamlines. In summary, these negative findings support our hypothesis by corroborating that functionally not lateralized brain systems such as the olfactory and hippocampal system do not show any differences in its type of connectivity.

### Cross-species comparison

**Figure 5:**
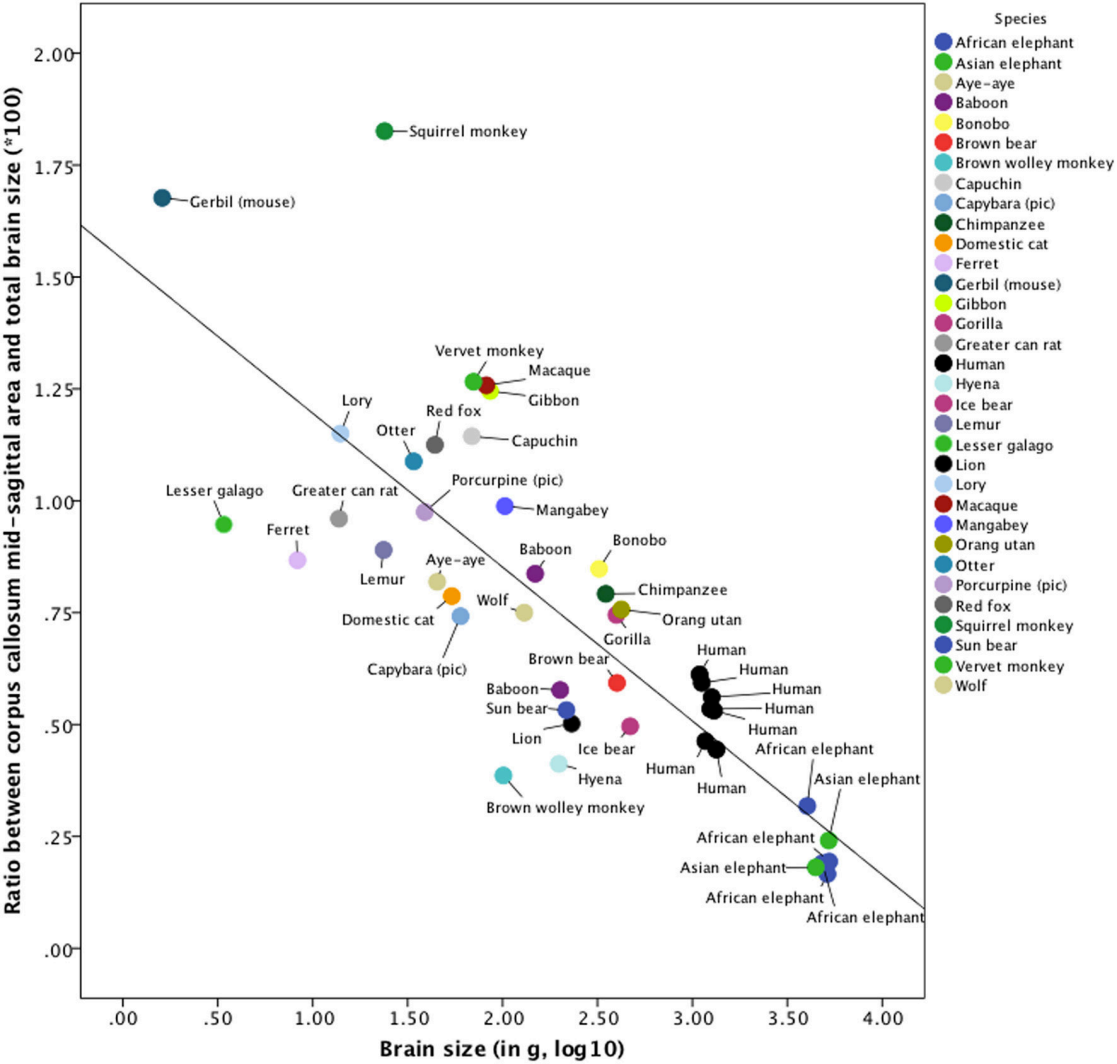
**Cross-species comparison of the ratio between corpus callosum mid-sagittal area and brain size**. The values used to construct this scatterplot as well as the references of the publications, from which these values were derived, can be found in Supplementary Table 4. Note that for humans, brain size was measured in cm^3^. The explained variance of this linear regression is *R*^2^ = 0.644.

The relevant indices of our cross-species comparison are listed in Supplementary Table 4. The ratio between corpus callosum mid-sagittal area and brain size decreases with increasing brain size. A non-parametric Kruskal-Wallis test indicated that these ratios are significantly different among elephants, humans, carnivores, and rodents [*n* = 44, χ^2^_(4)_ = 25.40, *p* = 0.00004, ϕ = 0.76]. Subsequent *post-hoc* Mann–Whitney *U*-tests revealed that the ratio in humans is significantly larger than that of the elephants (*n* = 13, *p* = 0.0012) and other primates (*n* = 24, *p* = 0.0005), but not significantly larger than the ratio of carnivores investigated (*n* = 17, *p* = 0.23). Due to the small sample sizes of these categories, results should be interpreted with caution and confirmed in future studies with more animals per species. The scatter plot of the brain sizes regressed against the ratios between corpus callosum mid-sagittal area and brain size is shown in Figure 5. The explained variance of this linear regression is *R*^2^ = 0.644.

### Validation analyses

We used connectivity data that are not prone to the sampling-related and distance-related tractography bias (Li et al., 2012) and were able to replicate our main findings based on DTI tractography data. In the first validation analysis based on T1-weighted MRI scans, the volume of the corpus callosum served as a measure of interhemispheric connectivity, whereas the remaining white matter volume served as a measure of intrahemispheric connectivity. A mixed ANOVA model (*N* = 138) revealed a highly significant interaction between brain size (small vs. large brains) and connectivity (interhemispheric vs. intrahemispheric connectivity) [*F*_(1, 136)_ = 161.4, *p* = 7.2E-25, η*_p_*^2^ = 0.54]. Subsequent *post-hoc t*-tests revealed highly significant and massively increased intrahemispheric connectivity in larger compared with smaller brains [*t*_(136)_ = 12.66, *p* = 9.5E-25, *d* = 2.17], whereas interhemispheric connectivity was also increased in larger compared with smaller brains, but with a moderate effect size [*t*_(136)_ = 2.77, *p* = 0.006, *d* = 0.48]. The connectivity ratio based on the volume of the corpus callosum divided by the remaining white matter volume also correlated inversely with brain size (*r* = −0.406, *p* = 4.0E-7, one-tailed, Pearson's correlation).

The second validation analysis based on a deterministic streamline algorithm with a second order Runge-Kutta integration scheme that is less prone to the distance-related tractography bias. A mixed ANOVA model (*N* = 138) revealed a highly significant interaction between brain size (small vs. large brains) and connectivity (interhemispheric vs. intrahemispheric connectivity) [*F*_(1, 136)_ = 106.8, *p* = 7.9E-19, η*_p_*^2^ = 0.44]. Subsequent *post-hoc t*-tests revealed highly significant and massively increased intrahemispheric connectivity in larger compared with smaller brains [*t*_(136)_ = 10.65, *p* = 1.3E-19, *d* = 1.83], whereas interhemispheric connectivity was also increased in larger compared with smaller brains, but with a moderate effect size [*t*_(136)_ = 3.91, *p* = 0.0001, *d* = 0.67]. The connectivity ratio based on the number of interhemispheric streamlines divided by the number of intrahemispheric streamlines correlated inversely with brain size (*r* = −0.188, *p* = 0.014, one-tailed, Pearson's correlation).

### Summary of results

There is a highly significant interaction between brain size (small vs. large brains) and the type of connectivity (interhemispheric vs. intrahemispheric connectivity) across the whole sample. The same interactions can be found when comparing small vs. large female brains and small vs. large male brains. Intrahemispheric connectivity was massively increased in larger compared with smaller human brains (Cohen's *d* = 1.65), whereas interhemispheric connectivity tended to be only slightly increased in larger compared with smaller brains (Cohen's *d* = 0.45). Brain size explains 59% of the variance in intrahemispheric connectivity, whereas only 9% of the variance in interhemispheric connectivity is explained by brain size. In addition, brain size was inversely correlated with the connectivity ratio of interhemispheric to intrahemispheric connectivity. The analysis of the hippocampal and anterior commissure served as a negative control condition and indeed revealed negative findings. The hypothesis of neuronal interconnectivity as a function of brain size can be applied also to non-human animals as revealed by our cross-species comparison. Additionally, we showed that the pattern of connectivity we reported in the present study is not biased by the sampling-related and distance-related tractography bias inherent in DTI fiber tractography.

## Discussion

We demonstrated, as predicted 20 years ago by Ringo and colleagues and in line with existing literature (Ringo, 1991; Ringo et al., 1994; Jäncke et al., 1997; Rilling and Insel, 1999a,b; Im et al., 2008; Leonard et al., 2008), that there is a significant interaction between brain size and the type of connectivity. This was achieved using DTI based quantitative fiber tractography in humans. To the best of our knowledge, we showed for the first time that the magnitude of the different types of connectivity (interhemispheric and intrahemispheric) in humans is subject to absolute brain size. In contrast to a recently published study that claimed for a sexual dimorphism in the human structural connectome in the form of increased interhemispheric connectivity in women and increased intrahemispheric connectivity in men (Ingalhalikar et al., 2014), we provide strong evidence that this pattern of connectivity is driven by brain size and not by sex *per se* due to the confounding of sex and brain size.

### Brain size vs. gender

Although gender and brain size are highly correlated, the hypothesis of neuronal interconnectivity as a function of brain size is independent of gender. As expected, brain size and gender were significantly related in our sample as well (Cohen's *d* = 1.52); hence the large brain group is biased toward men (55/14 m/f), whereas the small brain group is biased toward women (55/14 f/m). However, we were able to replicate the effect found in the gender-pooled sample (*N* = 138) within the gender-specific subsamples (*n* = 69 each). The interaction within the gender-specific subsamples (35 small vs. 34 large brains) is statistically significant between small and large female brains as well as between small and large male brains. Therefore, the hypothesis of neuronal interconnectivity as a function of brain size is gender-independent and almost exclusively driven by brain size. Further support for the fact that the important factor, which drives the connectivity pattern under investigation, is brain size and not gender, can be derived from our cross-gender subgroup comparisons. These comparisons revealed stronger effect sizes when comparing small female brains with large male brains (larger brain size difference) on one hand, and no effect when comparing large female with small male brains (no brain size difference) on the other hand.

However, in a recently published study it has been suggested that women have increased interhemispheric connectivity and men have increased intrahemispheric connectivity (Ingalhalikar et al., 2014), whereas the findings of the present study clearly show that this pattern of connectivity can also be found within genders when comparing small-brained with large-brained women and small-brained with large-brained men (see Figure 2). Therefore, the effects reported by Ingalhalikar and colleagues are, in our opinion, caused by differences in brain size and not by gender as suggested by the authors (Ingalhalikar et al., 2014). To directly disprove their conclusions we first replicated their finding in a random subsample of our study (27 women vs. 27 men). These groups significantly differed in brain size [*t*_(52)_ = −6.37, *p* = 5.0E-8, *d* = 1.77]. In line with their results (Ingalhalikar et al., 2014) we found an interaction between sex and type of connectivity [*F*_(1, 52)_ = 18.1, *p* = 0.00009, η*_p_*^2^ = 0.26]. *Post-hoc t*-tests revealed strongly increased intrahemispheric connectivity in male compared with female brains [*t*_(52)_ = 3.04, *p* = 0.0037, *d* = 0.84], whereas interhemispheric connectivity was slightly increased in female brains, but did not reach statistical significance [*t*_(52)_ = −1.27, *p* = 0.21, *d* = −0.35]. To investigate sex differences independent of brain size, we then formed a female (*n* = 27) and a male (*n* = 27) subgroup that were almost perfectly matched for brain size [*t*_(52)_ = 0.008, *p* = 0.99]. In groups with equal brain sizes, no significant interaction between sex and type of connectivity was found [*F*_(1, 52)_ = 0.008, *p* = 0.93]. *T*-tests showed that neither intrahemispheric connectivity is increased in men [*t*_(67)_ = −0.08, *p* = 0.94] nor is interhemispheric connectivity increased in women [*t*_(67)_ = −0.54, *p* = 0.59]. Our findings provide strong evidence against the conclusions drawn by Ingalhalikar et al. (2014) and clearly show that this apparent sex difference is merely driven by difference in brain size. Further support for our conclusions can be derived from a recently published study that showed that individual differences in brain size account for apparent sex differences in the anatomy of the human corpus callosum (Luders et al., 2014). However, at least for GM volumes it has been shown that even when controlling for brain size between women and men, there are still remaining differences in local GM volume that are related to gender (Luders et al., 2009).

### Allometry of brain connectivity

Our results indicate that with increasing brain size, the increase in the number of interhemispheric connections does not keep pace with the increase in the number of intrahemispheric connections. This suggests that the connectivity pattern as a function of brain size does not follow linear rules, a fact that has already been reported for other than connectomic anatomical brain measurements (Im et al., 2008). As brain size increases, the cortex increased only slightly, but the degree of sulcal convolution increases dramatically, indicating that human cortices are not simply scaled versions of one another (Im et al., 2008) as it was the case for the human connectome in the present study. The results reported by Im and colleagues are consistent with the hypothesis of neuronal interconnectivity as a function of brain size by suggesting that greater local clustering of interneuronal connections would be required in a larger compared with a smaller brain (Ringo, 1991; Ringo et al., 1994; Anderson, 1999).

First suggested by Ringo et al. (Ringo, 1991; Ringo et al., 1994), the hypothesis of neuronal interconnectivity as a function of brain size cannot be rejected, at least for the human species. It seems really to be the case that larger brains compensate their increased conduction delays in transcallosal information transfer by not increasing the local information processing capacity that depends on the corpus callosum, but by over-proportionally increasing the intrahemispheric amount of fibers that interconnect these local processing units.

Our findings are also in line with the hypothesis that fiber tension between local cortical areas would induce cortical folds (Van Essen, 1997; Herculano-Houzel et al., 2010). Two studies published (Nonaka-Kinoshita et al., 2013; Stahl et al., 2013) might provide evidence for the tension-based theory of morphogenesis and compact wiring in the central nervous system (Van Essen, 1997). One study investigated the DNA-associated protein TRNP1, which regulates tangential and radial expansion of the cortex, by using gain- and loss-of-function experiments in the mouse cerebral cortex *in vivo* and this study demonstrated that high TRNP1 levels promote neural stem cell self-renewal and tangential expansion, whereas lower levels promote radial expansion, with a potent increase of the number of intermediate progenitors and basal radial glial cells leading to folding of the otherwise smooth murine cerebral cortex (Stahl et al., 2013). The other study reported that the controlled expansion of uni-potent basal progenitors in mouse embryos led to megalencephaly, with increased cortical surface area, but not to cortical folding. In contrast, expansion of multipotent basal progenitors in the naturally gyrencephalic ferret was sufficient to drive the formation of additional folds and fissures. In both models, changes occurred while preserving a structurally normal, six-layered cortex (Nonaka-Kinoshita et al., 2013). We can imagine that such mechanisms might also promote intrahemispheric over interhemispheric connectivity with increasing brain size, but this is highly speculative. Further research is needed using different methodological approaches.

Further support for the hypothesis of neuronal interconnectivity as a function of brain size can be derived from an investigation showing that the lengths of transcallosal fibers are negatively correlated with the callosal cross-sectional area containing these fibers in four (the exception was the callosal isthmus) of the five investigated callosal subregions (Lewis et al., 2009). This negative relationship implies that a larger callosal mid-sagittal area goes with a shorter transcallosal fiber length and the length of these connections, on average, accounted for about 25.5% of the variance in degree of interhemispheric connectivity (Lewis et al., 2009). The cross-sectional area of the CC is a proxy measure for interhemispheric connectivity and it has previously been shown that its relative (not absolute) size is inversely related to forebrain volume (Jäncke et al., 1997) and whole cerebral volume (Leonard et al., 2008). However, it is important to note that *absolute* CC volume is increased in larger compared with smaller brains, but the *relative* CC volume (the absolute CC volume divided by the ICV or the TBV) is increased in smaller compared with larger brains. Similarly, regressing absolute CC volume against brain size results in a positive correlation, whereas regressing relative CC volume against brain size results in a negative correlation, illustrating the under-proportional increase of the absolute CC volume with increasing brain size (Jäncke et al., 1997; Leonard et al., 2008). The negative correlation between absolute CC area and the length of its fibers (Lewis et al., 2009) fits well with the predictions that the hypothesis of neuronal interconnectivity as a function of brain size provides, e.g., that interhemispheric connectivity is reduced in larger compared with smaller brains and hence the finding by Lewis and colleagues is in accordance with the findings of the present study (Lewis et al., 2009).

We also analyzed another relative connectivity measure, i.e., the ratio of the interhemispheric connectivity measure to the intrahemispheric connectivity measure and associated these measures with brain size. These correlations revealed inverse relations between brain size and the connectivity ratio in the gender-pooled sample as well as within the gender-specific subsamples. Only the correlation in the gender-pooled sample reached statistical significance. However, as shown in Figure 4, the smallest and largest brain investigated in the present study showed a positive correlation between TBV and the connectivity ratio. This finding does not fit well with our predictions; nevertheless, we avoid speculating about the possible reasons of this unexpected result.

### Hemispheric lateralization of brain functions

On a functional level, differences in interhemispheric transfer times (IHTTs) between women and men measured with electroencephalographic event-related potentials have been reported. At least two studies showed that IHTTs were increased in men compared with women (Nowicka and Fersten, 2001; Moes et al., 2007), verifying that IHTTs are indeed prolonged in larger compared with smaller human brains supporting the conjecture made by Ringo and colleagues (Ringo, 1991; Ringo et al., 1994). In addition, IHTTs were more asymmetrical (with respect to the hemifield of stimulus presentation) in the larger-brained men than in the smaller-brained women, supporting the prediction, originally proposed by Ringo and colleagues 20 years ago (Ringo, 1991; Ringo et al., 1994), that larger brains should show increased cortical specialization and a stronger hemispheric lateralization of brain functions than smaller brains (see below).

The differences in the connectomic architecture revealed between large and small brains in the present study should have consequences on the implementation of brain functions. One such consequence, as already stated by Ringo et al. (Ringo, 1991; Ringo et al., 1994), might be the stronger hemispheric lateralization of brain functions in larger brains (more frequent in men) compared with smaller brains (more frequent in women). Indeed, there is strong empirical evidence that visual and auditory processing are stronger lateralized in men than in women (Hiscock et al., 1994, 1995) and women being less lateralized than men especially for the faculty of language (Shaywitz et al., 1995), although there are also contradictory findings (Kaiser et al., 2009). Furthermore, it has been shown more directly by using brain size instead of gender that the smaller the brain the smaller the functional leftward asymmetry for language processing (Josse et al., 2006; Tzourio-Mazoyer et al., 2010). It has also been shown that the planum temporale, associated with higher auditory processing, is more asymmetrically organized in men compared with women (Kulynych et al., 1994). Again, a more lateralized language system in men (larger brains) compared with women (smaller brains) is in good accordance with the predictions proposed by Ringo and colleagues 20 years ago (Ringo, 1991; Ringo et al., 1994).

The hypothesis of neuronal interconnectivity as a function of brain size is also compatible with findings published by Galaburda and Geschwind about 30 years ago. It has been shown that the magnitude, if not also the direction, of cortical asymmetry predicts the relative numbers of neurons comprising a given pair of hemispheric architectonic homologs such that the more asymmetric the region is, the smaller the number of neurons (Geschwind and Galaburda, 1985a,b,c; Galaburda et al., 1990). Similarly, the more asymmetric a region is, the smaller the density of interhemispheric connections and (probably) the greater the density of intrahemispheric connections. In general, more symmetrical brains appear to have a larger corpus callosum and thus showed increased interhemispheric connectivity (Geschwind and Galaburda, 1985a,b,c; Galaburda et al., 1990; Aboitiz et al., 1992c; Dorion et al., 2000). For example, fewer callosal projections between the plana temporales were found when their minicolumn spacing was more asymmetrical (Chance et al., 2006) and there is a correlation between the magnopyramidal neuron density in the planum temporale and axon number in the isthmus of the corpus callosum and this correlation seems to be absent in schizophrenia (Simper et al., 2011). Therefore, the hypothesis of neuronal interconnectivity as a function of brain size can be related to the symmetry / asymmetry of cortical brain regions and might also account for the repeatedly observed stronger lateralization of brain functions in men. But, investigations on subjects who showed an atypical functional lateralization, i.e., both language and spatial processing are lateralized in the right hemisphere, revealed increased fractional anisotropy in callosal connections suggesting rather enhanced than reduced interhemispheric connectivity (Häberling et al., 2011). However, when considering an atypical lateralization as a type of reduced typical lateralization, enhanced interhemispheric would fit nicely with the prediction made by the hypothesis of neuronal interconnectivity as a function of brain size.

In contrast to cortical systems, which are heavily lateralized in human subjects, the olfactory and hippocampal system do not show any functional lateralization and therefore the anterior and hippocampal commissure have been used as negative control regions. The number of interhemispheric streamlines between both primary olfactory cortices (part of the anterior commissure) and those between both hippocampi (hippocampal commissure) were not significantly different between small and large brains, enhancing the specificity of our findings.

### Is the hypothesis restricted to humans?

The hypothesis of neuronal interconnectivity as a function of brain size seems not to be restricted to humans because it is further supported by experimental evidence derived from animal studies. Across different species (human, horse, cow, dog, cat, rabbit, and rat) the proportion of callosal fibers in relation to brain size or to the estimated number of cortical cells decrease with increasing brain mass (Olivares et al., 2001), thereby reducing the degree of interhemispheric connectivity. More recently, Herculano-Houzel and colleagues showed mathematically that the proportion of cortical neurons connected through white matter decreases in larger primate brains compared with smaller ones (Herculano-Houzel et al., 2010).

In order to test the hypothesis that hemispheric lateralization evolved as a consequence of reduced interhemispheric connectivity, Hopkins and Rilling investigated whether neuromorphometric asymmetries were associated with variation in the ratio of CC size to TBV and to neocortical surface area in human and non-human primates (Hopkins and Rilling, 2000). Magnetic resonance images were collected from a sample of 45 primates including new world monkeys, old world monkeys, lesser apes, great apes, and humans. Results indicated that brain asymmetry significantly predicts the ratio of CC size to TBV and to neocortical surface area. Subsequent analyses revealed that species variation in functional asymmetries in the form of handedness are also inversely related to the ratio of CC size to neocortical surface area. These results support the hypothesis that brain asymmetries (leftward in the context of language and handedness) may have evolved as a consequence of reduced interhemispheric connectivity (Hopkins and Rilling, 2000). A similar finding was reported for the Wistar rat (Rosen et al., 1989).

Our results are also in accordance with observations that the cortex in rat, which is relatively small, is not specialized at all (Lashley, 1950; Meyer, 1984), whereas monkeys, which have large brains, have a very specialized cortex (Van Essen and Maunsell, 1983; Maunsell and Newsome, 1987) and neurological evidence from humans indicates cortical specialization and hemispheric lateralization. In general, those investigating cortical organization report a positive correlation between brain size and the number of distinct cortical areas (Campos and Welker, 1976; Kaas, 1987).

### Cross-species comparison

It follows from the hypothesis of neuronal interconnectivity as a function of brain size that the larger the brain, the more functionally lateralized it should be. If true not only for the humans, one would expect elephant and cetacean (whales) brains to be the most functionally lateralized of all (even more lateralized than our own). Indeed, there is evidence, at least on the behavioral level, that elephants and whales show strongly lateralized behaviors. With respect to trunk movements in elephants there exist “right-trunkers” and “left-trunkers” and in whales, feeding, hunting, and social behaviors are lateralized too (Clapham et al., 1995; Marino and Stowe, 1997; Martin and Niemitz, 2003; Haakonsson and Semple, 2009; Karenina et al., 2010; Siniscalchi et al., 2012).

However, to the best of our knowledge, there is no literature with respect to lateralization in elephants and whales on the neural level. Nevertheless, our cross-species comparison of the ratio between mid-sagittal corpus callosum area and brain size (Figure 5 and Supplementary Table 4) suggested that the hypothesis of neuronal interconnectivity as a function of brain size is not restricted to humans, but might represent a general organization principle of biology applying also to non-human animals.

### Limitations

Several limitations of the present work are worth mentioning. First, one might argue that our main findings could also be explained by two well-known biases inherent to whole-brain streamline tractography; the sampling-related and the distance-related bias (Zalesky, 2008; Zalesky and Fornito, 2009; Li et al., 2012). Although both validation analyses clearly shows that our findings are not affected by these two tractography biases, we nevertheless discuss the potential influences of these biases here.

The sampling-related bias postulates that larger brains comprise more white-matter volume and as a consequence also contains more seed voxels and therefore a larger brain will comprise more streamlines simply owing to the fact that more streamlines were initialized in larger compared with smaller brains. Due to the fact that the additional voxels in larger brains are equally distributed across the whole brain, the sampling-related bias should affect interhemispheric and intrahemispheric connectivity in a similar way. Further, the fact that long-distance fiber bundles occupy more volume and are thus sampled by a greater number of streamlines is not in favor of our hypothesis because callosal fibers belong the longest ones in the human brain, but the longest fibers are additionally prone to the distance-related bias (see below).

The distance-related bias postulates that long-distance fibers are more difficult to track with DTI than short-distance fibers (Zalesky, 2008; Zalesky and Fornito, 2009; Li et al., 2012). Due to the fact that long-distance fibers are more abundant in the corpus callosum compared with intrahemispheric connections, our interhemispheric connectivity measure would be more biased than our intrahemispheric connectivity measure. However, similar differences as those found in the DTI analysis have been observed when using the mid-sagittal area or volume of the corpus callosum as a proxy measure of interhemispheric connectivity that is not prone to the distance-related tractography bias. Furthermore, the sampling-related and distance-related bias counteract and hence there is the possibility that the two biases might tend to cancel out each other. In addition, the DTI-based validation analysis that used a second order Runge-Kutta integration scheme, which is less prone to the distance-related bias, confirmed the findings of the main DTI analysis as well as those of the other validation analysis based on T1-weighted MRI data.

Second, the operationalization of connectivity in the present study is based on the number of reconstructed streamlines from DTI data, which has a poor spatial resolution in relation to the real size of single axons and small axonal bundles. Increased numbers of reconstructed streamlines between two groups can result from the same number of axons in both groups, but axonal diameters are increased in one relative to the other group, or conversely, the axons are of the same diameters in both groups, but more abundant in one compared to the other group. However, both constellations can be interpreted rather as a sign of increased than reduced neuronal interconnectivity.

Third, the smallest and largest brains investigated in the present study did not follow entirely our predictions, suggesting that in extremely small and large brains other factors than brain size *per se* might also play an important role in determining the ratio of interhemispheric to intrahemispheric connectivity. Forth, the lack of significant differences in the number of hippocampal and anterior commissural connections between large and small brains rather support, in our opinion, the hypothesis of neuronal interconnectivity as a function of brain size than contradict it, because it is not yet convincingly shown that the hippocampal and olfactory system is lateralized in humans.

Last, although we showed an interaction between brain size and the connectivity pattern in the adult human brain, we unfortunately do not know when in life and how these differences manifest. It is also unclear whether nature or nurture is more important in determining the connectivity pattern, what the mechanisms are during axonal path finding, and which roles apoptosis and axonal pruning actually play. It remains unknown whether the connectivity differences are implemented in embryonic and fetal development or in postnatal and maturational periods, and to what extent these connections are modifiable by neuroplastic changes during childhood and adulthood. It is further unclear whether the mechanisms involved in implementing the observed connectivity pattern are active processes during axonal path finding periods or passive processes based on apoptosis or environmental-driven pruning or the result of both processes. However, such questions cannot be answered in the present study and needs further investigations using other methodological approaches in the relevant animal models.

## Conclusion

As predicted 20 years ago by Ringo and colleagues the hypothesis of neuronal interconnectivity as a function of brain size seems to be confirmed, at least for humans. The hypothesis suggests that larger brains compensate their increased delay in transcallosal information transfer by reducing interhemispheric connectivity and enhancing intrahemispheric connectivity. As a result of this, larger brains show increased cortical specialization and stronger hemispheric lateralization than smaller brains. We showed that there is a significant interaction between brain size and interhemispheric vs. intrahemispheric connectivity, favoring intrahemispheric over interhemispheric connectivity with increasing brain size. This effect is independent of gender. The hypothesis of neuronal interconnectivity as a function of brain size represents an organization principle of the human connectome and furthermore, as suggested by our cross-species comparison, a general organization principle of biology applying also to non-human animals.

## Author contributions

Jürgen Hänggi and Lutz Jäncke conceived the study and formulated the hypothesis. Laszlo Fövenyi, Franziskus Liem, Martin Meyer, and Jürgen Hänggi were involved in acquiring the data. Franziskus Liem, Laszlo Fövenyi, and Jürgen Hänggi processed and analyzed the data. Jürgen Hänggi and Lutz Jäncke drafted the first version of the manuscript. All authors were involved in the preparation of this manuscript and read and approved the final version of it.

### Conflict of interest statement

The authors declare that the research was conducted in the absence of any commercial or financial relationships that could be construed as a potential conflict of interest.
