# Supplementary material for: The hypothesis of neuronal interconnectivity as a function of brain size—a general organization principle of the human connectome
Source: Front Hum Neurosci. 2014 Nov 11;8:915. doi: 10.3389/fnhum.2014.00915 (PMC4227509; doi:10.3389/fnhum.2014.00915)
Supplement: Supplementary file 1 [file DataSheet1.DOC]

***Supplementary Material***

**The hypothesis of neuronal interconnectivity as a function of brain size – A general organization principle of the human connectome**

Jürgen Hänggi^1*^, Laszlo Fövenyi^1^, Franziskus Liem^1,2^, Martin Meyer^2^, Lutz Jäncke^1,3-6*^

^1^ Division Neuropsychology, Institute of Psychology, University of Zurich, Switzerland.

^2^ Research Unit for Neuroplasticity and Learning in the Healthy Aging Brain (HAB LAB), Institute of Psychology, University of Zurich, Switzerland.

^3^ International Normal Aging and Plasticity Imaging Center (INAPIC), University of Zurich, Switzerland.

^4^ Center for Integrative Human Physiology (ZIHP), University of Zurich, Switzerland.

^5^ University Research Priority Program (URPP), Dynamic of Healthy Aging, University of Zurich, Switzerland.

^6^ Department of Special Education, King Abdulaziz University, Jeddah, Saudi Arabia.

**1. Supplementary Introduction**

Our hypothesis was generated from the theoretical model about neuronal interconnections as a function of brain size proposed 20 years ago by Ringo and colleagues who postulated, based on considerations about conduction delays of information transfer and cellular costs, that maintaining absolute connectivity in larger compared with smaller brains would be computationally inefficient and expensive with respect to the brain mass devoted to these connections . These authors developed a computer program that simulates the brain’s inter- and intrahemispheric information processing and is based on a structural as well as a functional component. The structural component was composed of six modules, of which the two largest modules represent the two hemispheres (Supplementary Fig. 1).


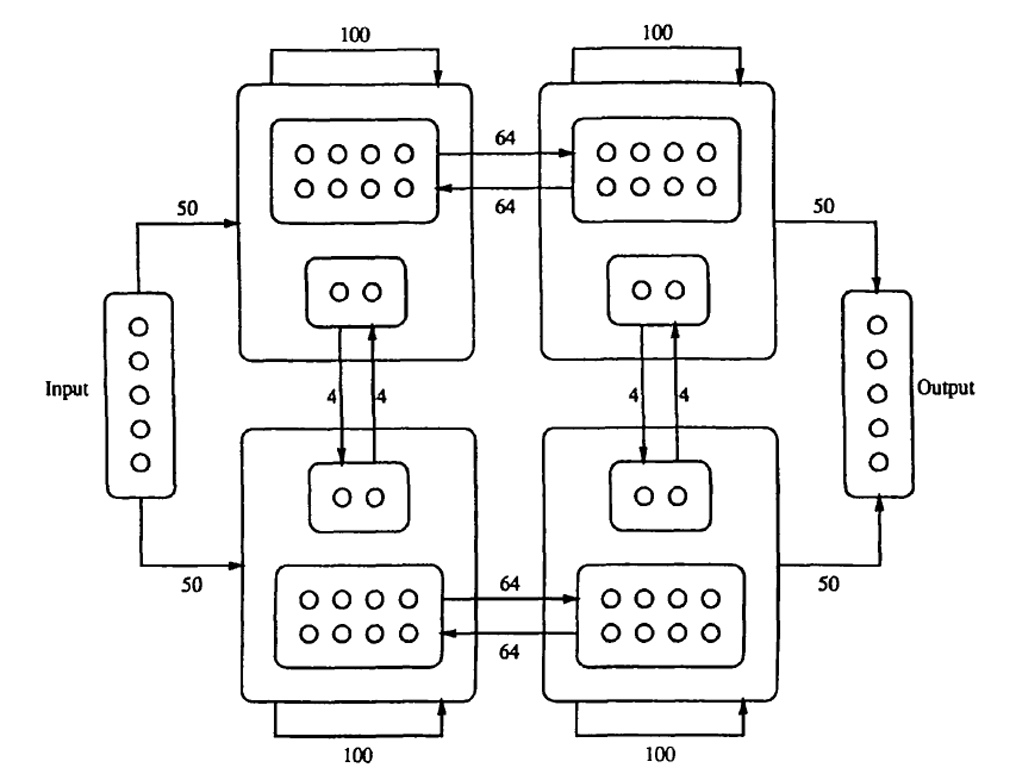


**Supplementary Figure 1.** The architecture of the neural network simulation. The small circles represent information-processing units and the boxes represent groupings of information-processing units. An arrow between two boxes means that all the units in the source box have a connection to all the units in the target box. The two large boxes (modules) in the upper part of the figure, with 10 units each (and two internal boxes), form one hemisphere. These modules are identical except for their inputs and outputs. Similarly, the two large boxes in the lower part of the figure form the other hemisphere. The value listed with each arrow is the number of connections between corresponding boxes. The connections between the two hemispheres are sparse. Two units in each of the two modules within a hemisphere are interconnected with two of the units in the corresponding module on the other side. The interhemispheric connections have a delay of 10 time steps in one condition (large brains) and one time step in the other condition (small brains). All other connections have a delay of one time step in both conditions. The figure is adapted from the original publication by Ringo and colleagues . Reprint with the permission from Oxford University Press.

Three Hundreds and twenty-eight connections within one hemisphere simulated the intrahemispheric connectivity among 20 information-processing units (represented by circles). The two hemispheres were connected by 16 connections organized in four modules each module containing two information-processing units that simulated the information transfer of the corpus callosum (CC) . The CC, the main fibre tract connecting the two hemispheres, consists of approximately 200-350 million fibres in humans . The question how does the CC mediate interhemispheric transfer and how transcallosal transfer of information is related to functional brain asymmetries is not discussed here but are addressed in the following review articles . In the computer model of Ringo and colleagues, the number of interhemispheric connections is 2.4 % of all connections and this fraction is in line with findings in the rhesus monkey suggesting that only 1-3 % of all neurons are connected via the CC . The data needed for processing in the functional component (see below) were inputted by 50 connections per hemisphere with the use of the input module and after processing, results were outputted by 50 connections per hemisphere with the use of the output module (Supplementary Fig. 1).

The functional component of the model was implemented as a software tool that is able to learn (adapt to) a specific pattern recognition/comparison task. The task was to compare 10 randomly generated five-digit number arrays consisted of +1 and -1 with other 10 randomly generated five-digit number arrays consisted of +1 and -1 and to indicate how many of the five-digit number arrays were equal. This kind of task was executed under two different conditions. Both conditions were equal except the time that is needed for the information transfer between the two hemispheres (callosal delay). In one condition, interhemispheric information transfer needed 10 time steps, whereas only one time step is needed in the other condition. The implementation of two different callosal transmission times allowed the authors to simulate large (longer transmission times) and small brains (shorter transmission times) .

In both conditions, the adaptive simulation program was initially trained with 50 runs in order to optimize the pattern comparison task. After these training runs, the interhemispheric connections were cut in both conditions and the percent correct comparisons after 15, 20, 25, 30, 35, 40, 45, 50 and 75 processing steps were counted, which determine the allowed computation time (called output time in Supplementary Fig. 2).


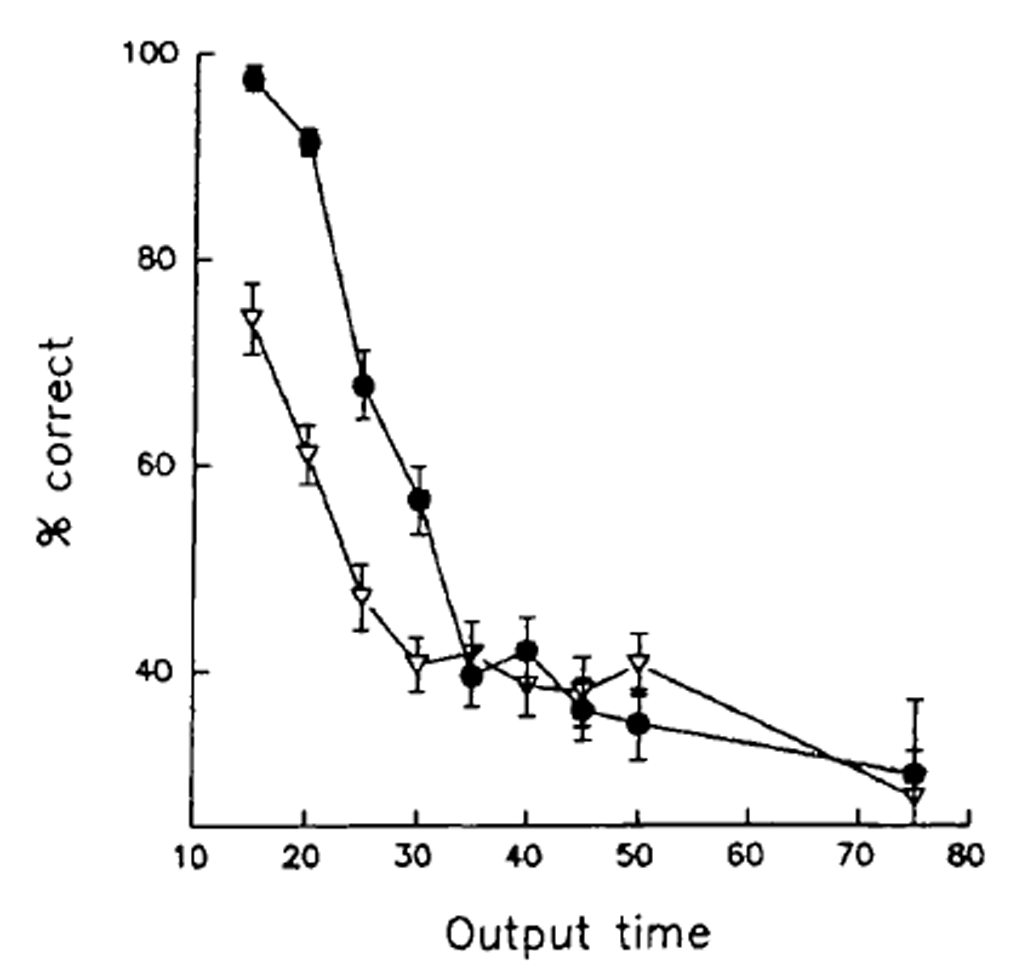


**Supplementary Figure 2.** The plot shows the performance of the networks following disconnection of the interhemispheric connections. For output times below 35 time steps, the network with the fast interhemispheric link (small brains, open triangles) is clearly more disrupted by the disconnection than the network with a slow interhemispheric link (large brains, solid circles). For long output times the networks do not differ. The error bars cover ± one standard error of the mean. The variability was due to the random initial assignment of the connection weights. The figure is adapted from the original publication by Ringo and colleagues . Reprint with the permission from Oxford University Press.

Supplementary Figure 2 shows the results of these simulations and one can see that the network with longer callosal transmission time (larger brains) was less impaired after disconnecting the hemispheres and therefore produced more correct comparisons in the range of 15 to 35 processing steps compared with the network with shorter callosal transmission time (smaller brains) .

Ringo and colleagues concluded that hemispheres within a network with longer callosal transmission times (i.e. larger brains) work rather independently from each other compared to hemispheres within a network with shorter callosal transmission times (i.e. smaller brains); therefore, smaller brains should be more strongly connected interhemispherically, whereas larger brains should show increased intrahemispheric connectivity compared with smaller brains .

**2. Supplementary Results**

In the supplementary results, we first show the demographic data, global brain measures, and connectomic characteristics derived from the 90 ROIs connectivity matrix of the gender-separated groups. Subsequently, the results of the connectivity measures that were derived from the 180 ROIs connectivity matrix of the gender-separated groups are presented (see below).

| **Supplementary Table 1** |  |  |  |  |  |  |  |  |  |
| --- | --- | --- | --- | --- | --- | --- | --- | --- | --- |
|  | **Small female brains (n = 35)** | | | | **Error probability** | **Large female brains (n = 34)** | | | |
| **Demographic and global**  **brain measures** | **Mean** | **SD** | **Minimum** | **Maximum** | **p-value** | **Mean** | **SD** | **Minimum** | **Maximum** |
| Age (years) | 26.4 | 6.80 | 19.0 | 50.0 | 0.92 | 26.3 | 7.40 | 18.0 | 57.0 |
| Handedness (left/right) | 11/24 | - | - | - | 0.86 | 10/24 | - | - | - |
| Total brain volume (ccm) | 1,046.7 | 52.5 | 858.7 | 1,114.2 | **< 0.001** | 1,176.2 | 39.5 | 1,117.5 | 1,277.4 |
| Total gray matter volume (ccm) | 616.9 | 32.1 | 503.6 | 672.2 | **< 0.001** | 682.4 | 32.0 | 622.1 | 742.6 |
| Total white matter volume (ccm) | 429.8 | 30.3 | 355.1 | 489.7 | **< 0.001** | 493.8 | 24.7 | 430.9 | 548.7 |
| **Connectivity measures (90 nodes)** |  |  |  |  |  |  |  |  |  |
| Total number of streamlines | 2,314,080 | 251,312 | 1,824,454 | 2,891,695 | **< 0.001** | 2,596,166 | 209,352 | 2,325,539 | 3,134,647 |
| Streamlines omitted | 1,309,227 | 133,671 | 1,034,426 | 1,611,036 | **< 0.001** | 1,438,696 | 92,473 | 1,272,071 | 1,678,308 |
| Streamlines used to populate matrix | 1,004,853 | 129,884 | 790,028 | 1,341,216 | **< 0.001** | 1,157,471 | 132,015 | 960,300 | 1,508,733 |
| Selfloops | 628,016 | 95,178 | 482,696 | 834,808 | **< 0.001** | 722,926 | 90,765 | 590,584 | 931,568 |
| Interhemispheric streamlines | 127,536 | 29,015 | 74,355 | 189,831 | **0.04** | 141,189 | 24,348 | 97,588 | 192,938 |
| Intrahemispheric streamlines (left and right) | 563,309 | 66,461 | 460,073 | 751,275 | **< 0.001** | 654,819 | 74,422 | 543,300 | 862,548 |
| Connectivity ratio | 0.226 | 0.041 | 0.132 | 0.316 | 0.25 | 0.216 | 0.029 | 0.158 | 0.271 |
|  |  |  |  |  |  |  |  |  |  |

**Supplementary Table 1. Demography, global brain measures, and connectomic characteristics of the small and large brain group in the female subsample (n = 69). Connectivity ratio was calculated by dividing the interhemispheric by the intrahemispheric connectivity measure. The difference in handedness was determined by a χ^2^-test and all other differences were determined by t-tests for independent samples with 67 degrees of freedom. n, number of subjects; SD, standard deviation.**

| **Supplementary Table 2** |  |  |  |  |  |  |  |  |  |
| --- | --- | --- | --- | --- | --- | --- | --- | --- | --- |
|  | **Small male brains (n = 35)** | | | | **Error probability** | **Large male brains (n = 34)** | | | |
| **Demographic and global**  **brain measures** | **Mean** | **SD** | **Minimum** | **Maximum** | **p-value** | **Mean** | **SD** | **Minimum** | **Maximum** |
| Age (years) | 24.6 | 4.42 | 17.4 | 37.9 | 0.76 | 24.9 | 4.75 | 20.2 | 42.8 |
| Handedness (left/right) | 13/22 | - | - | - | 0.68 | 11/23 | - | - | - |
| Total brain volume (ccm) | 1,166.2 | 60.3 | 987.1 | 1,239.4 | **< 0.001** | 1,320.0 | 55.5 | 1,243.0 | 1,446.5 |
| Total gray matter volume (ccm) | 685.2 | 37.9 | 570.2 | 743.2 | **< 0.001** | 760.5 | 37.9 | 570.2 | 743.2 |
| Total white matter volume (ccm) | 481.0 | 28.6 | 417.0 | 543.3 | **< 0.001** | 559.5 | 30.6 | 511.4 | 645.9 |
| **Connectivity measures (90 nodes)** |  |  |  |  |  |  |  |  |  |
| Total number of streamlines | 2,581,420 | 283,973 | 2,088,707 | 3,196,590 | **< 0.001** | 2,869,184 | 229,468 | 2,506,539 | 3,451,223 |
| Streamlines omitted | 1,457,526 | 156,049 | 1,166,301 | 1,759,266 | **< 0.001** | 1,575,676 | 118,724 | 1,419,860 | 1,862,649 |
| Streamlines used to populate matrix | 1,123,894 | 138,947 | 909,486 | 1,478,607 | **< 0.001** | 1,293,508 | 129,968 | 1,047,682 | 1,598,552 |
| Selfloops | 709,326 | 114,905 | 519,508 | 986,812 | **< 0.001** | 828,626 | 97,965 | 688,676 | 1,062,856 |
| Interhemispheric streamlines | 131,294 | 26,497 | 80,747 | 207,150 | 0.15 | 141,422 | 31,589 | 71,994 | 207,746 |
| Intrahemispheric streamlines (left and right) | 637,937 | 69,235 | 517,569 | 818,683 | **< 0.001** | 737,773 | 64,876 | 621,762 | 883,385 |
| Connectivity ratio | 0.206 | 0.033 | 0.142 | 0.273 | 0.08 | 0.191 | 0.036 | 0.114 | 0.255 |
|  |  |  |  |  |  |  |  |  |  |

**Supplementary Table 2.** Demography, global brain measures, and connectomic characteristics of the small and large brain group in the male subsample (n = 69). Connectivity ratio was calculated by dividing the interhemispheric by the intrahemispheric connectivity measure. The difference in handedness was determined by a χ^2^-test and all other differences were determined by t-tests for independent samples with 67 degrees of freedom. n, number of subjects; SD, standard deviation.

Here we report the results of the connectivity measures that were derived from the 180 ROIs connectivity matrix. The main purpose of these analyses was to ascertain that the number of parcellations does not bias the results of the connectivity measures derived from the 90 ROIs connectivity matrix that were reported in the main manuscript.

**2.1. Connectomic characteristics**

The connectomic characteristics derived from the 180 ROIs connectivity matrix of the gender-pooled sample (N = 138) are summarized in Supplementary Table 3.

| **Supplementary Table 3** |  |  |  |  |  |  |  |  |  |
| --- | --- | --- | --- | --- | --- | --- | --- | --- | --- |
|  | **Small brains (n = 69)** | | | | **Error**  **probability** | **Large brains (n = 69)** | | | |
| **Connectivity measures (180 nodes)** | **Mean** | **SD** | **Minimum** | **Maximum** | **p-value** | **Mean** | **SD** | **Minimum** | **Maximum** |
| Total number of streamlines | 2,397,895 | 246,429 | 1,824,454 | 3,003,328 | **< 0.001** | 2,778,401 | 250,490 | 2,303,657 | 3,451,223 |
| Streamlines omitted | 1,353,562 | 130,735 | 1,034,426 | 1,685,013 | **< 0.001** | 1,535,205 | 128,041 | 1,251,601 | 1,862,649 |
| Streamlines used to populate matrix | 1,044,332 | 130,320 | 790,028 | 1,425,252 | **< 0.001** | 1,243,196 | 138,335 | 1,024,760 | 1,598,552 |
| Selfloops | 432,223 | 77,124 | 318,052 | 646,102 | **< 0.001** | 522,398 | 84,078 | 389,682 | 758,022 |
| Interhemispheric streamlines | 141,749 | 25,280 | 90,120 | 199,351 | **< 0.001** | 157,652 | 26,008 | 101,571 | 216,345 |
| Intrahemispheric streamlines (left and right) | 686,472 | 77,465 | 530,193 | 903,049 | **< 0.001** | 824,345 | 83,767 | 677,663 | 1,004,522 |
| Connectivity ratio | 0.206 | 0.027 | 0.134 | 0.268 | **< 0.001** | 0.191 | 0.025 | 0.135 | 0.239 |
|  |  |  |  |  |  |  |  |  |  |

**Supplementary Table 3.** Connectomic characteristics of the 180 ROIs connectivity matrix of the small and large brain group in the gender-pooled sample (n = 138). Compare the results with those of Table 1 in the main manuscript. The connectivity ratio was calculated by dividing the interhemispheric by the intrahemispheric connectivity measure. Differences were determined by t-tests for independent samples with 136 degrees of freedom. SD, standard deviation.

**2.2. Interaction between interhemispheric and intrahemispheric connectivity**

First, a mixed analysis of variance (ANOVA) model (N = 138) of the data derived from the 180 nodes connectivity matrix revealed a highly significant interaction between brain size (between-subject factor; small versus large brains) and connectivity (within-subject factor; interhemispheric versus intrahemispheric connectivity) (F_(1,136)_ = 114.2, p = 9.9E-20, η_p_^2^ = 0.46) (Supplementary Fig. 3A, c.f. Fig. 2A in the main manuscript). Subsequent post hoc t-tests revealed highly significantly and massively increased intrahemispheric connectivity in large compared with small brains (t_(136)_ = -10.0, p = 4.4E-18, d = 1.72), whereas interhemispheric connectivity was also increased in large compared with small brains, but with a moderate effect size (t_(136)_ = -3.6, p = 0.0004, d = 0.62).

We additionally applied an ANCOVA model that corrects for age because the two groups differed slightly in age (26.8 vs. 24.3 years in the small and large brain group, respectively). This analysis replicated the finding reported above (F_(1,136)_ = 117.2, p = 4.7E-20, η_p_^2^ = 0.46). When using ANCOVAs that correct for age instead of post hoc t-tests (see above) intrahemispheric connectivity is still significantly and massively increased in larger compared with smaller brains (F_(1,135)_ = 103.1, p = 2.4E-18, η_p_^2^ = 0.43), whereas interhemispheric connectivity was increased in larger compared with smaller brains, but with a moderate effect size (F_(1,135)_ = 13.4, p = 0.0004, η_p_^2^ = 0.09).

**
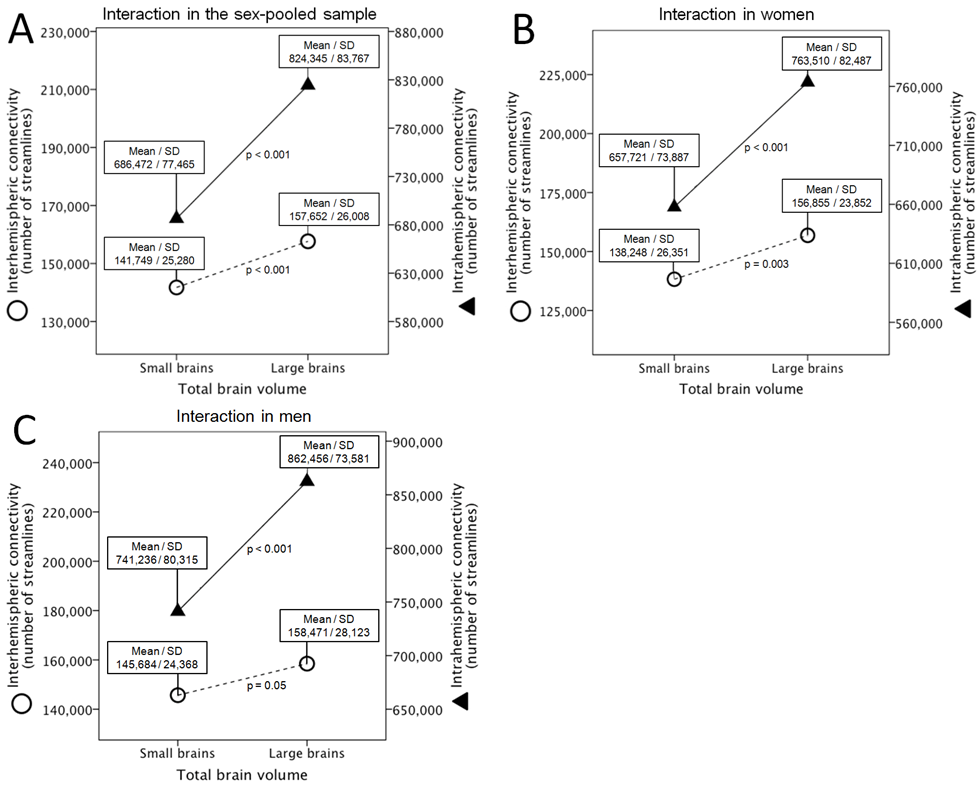
**

**Supplementary Figure 3.** Interaction between brain size and connectivity in the whole (A), in the female (B), and male sample (C). Data were derived from the 180 ROIs connectivity matrix. Compare the results with those of Fig. 2 in the main manuscript. Shown are analysis of variance models with large versus small brains as between-subject factor and *inter*hemispheric versus *intra*hemispheric connectivity as within-subject factor. Y-axes represent the number of reconstructed fibers.

Gender and TBV were not independent (t_(131.4)_ = -8.7, p = 1.1E-14, d = 1.52), therefore we applied a median split of the TBV within genders to rule out that the observed interaction is a gender instead a real brain size effect (Supplementary Table 4 and 5). Within these gender-specific samples, there were no significant differences with respect to age and handedness. As expected and intended by the median split, the small (n = 35) and large (n = 34) female brain group (Supplementary Table 4) as well as the small (n = 35) and large male (n = 34) brain group (Supplementary Table 5) significantly differed with respect to TBV, total GM volume and total WM volume.

| **Supplementary Table 4** |  |  |  |  |  |  |  |  |  |
| --- | --- | --- | --- | --- | --- | --- | --- | --- | --- |
|  | **Small female brains (n = 35)** | | | | **Error**  **probability** | **Large female brains (n = 34)** | | | |
| **Connectivity measures (180 nodes)** | **Mean** | **SD** | **Minimum** | **Maximum** | **p-value** | **Mean** | **SD** | **Minimum** | **Maximum** |
| Total number of streamlines | 2,314,080 | 251,312 | 1,824,454 | 2,891,695 | **< 0.001** | 2,596,166 | 209,352 | 2,325,539 | 3,134,647 |
| Streamlines omitted | 1,309,227 | 133,671 | 1,034,426 | 1,611,036 | **< 0.001** | 1,438,696 | 92,473 | 1,272,071 | 1,678,308 |
| Streamlines used to populate matrix | 1,004,853 | 129,884 | 790,028 | 1,341,216 | **< 0.001** | 1,157,471 | 132,015 | 960,300 | 1,508,733 |
| Selfloops | 417,769 | 79,659 | 318,052 | 602,074 | **0.003** | 474,213 | 71,388 | 366,664 | 646,102 |
| Interhemispheric streamlines | 138,248 | 26,351 | 90,120 | 199,351 | **0.003** | 156,855 | 23,852 | 108,860 | 204,570 |
| Intrahemispheric streamlines (left and right) | 657,721 | 73,887 | 530,193 | 858,769 | **< 0.001** | 763,510 | 82,487 | 644,460 | 1,002,780 |
| Connectivity ratio | 0.210 | 0.029 | 0.134 | 0.268 | 0.51 | 0.206 | 0.023 | 0.166 | 0.246 |
|  |  |  |  |  |  |  |  |  |  |

**Supplementary Table 4.** Connectomic characteristics of the 180 ROIs connectivity matrix of the small and large brain group in the female subsample (n = 69). Compare the results with those of Table 2 in the main manuscript. The connectivity ratio was calculated by dividing the interhemispheric by the intrahemispheric connectivity measure. Differences were determined by t-tests for independent samples with 67 degrees of freedom. SD, standard deviation.

| **Supplementary Table 5** |  |  |  |  |  |  |  |  |  |
| --- | --- | --- | --- | --- | --- | --- | --- | --- | --- |
|  | **Small male brains (n = 35)** | | | | **Error**  **probability** | **Large male brains (n = 34)** | | | |
| **Connectivity measures (180 nodes)** | **Mean** | **SD** | **Minimum** | **Maximum** | **p-value** | **Mean** | **SD** | **Minimum** | **Maximum** |
| Total number of streamlines | 2,581,420 | 283,973 | 2,088,707 | 3,196,590 | **< 0.001** | 2,869,184 | 229,468 | 2,506,539 | 3,451,223 |
| Streamlines omitted | 1,457,526 | 156,049 | 1,166,301 | 1,759,266 | **< 0.001** | 1,575,676 | 118,724 | 1,419,860 | 1,862,649 |
| Streamlines used to populate matrix | 1,123,894 | 138,947 | 909,486 | 1,478,607 | **< 0.001** | 1,293,508 | 129,968 | 1,047,682 | 1,598,552 |
| Selfloops | 473,948 | 93,219 | 359,538 | 719,540 | **0.001** | 545,162 | 78,817 | 441,088 | 758,022 |
| Interhemispheric streamlines | 145,684 | 24,368 | 110,248 | 216,345 | **0.05** | 158,471 | 28,123 | 101,571 | 216,245 |
| Intrahemispheric streamlines (left and right) | 741,236 | 80,315 | 600,454 | 937,083 | **< 0.001** | 862,456 | 73,581 | 713,359 | 1,004,522 |
| Connectivity ratio | 0.197 | 0.025 | 0.154 | 0.251 | **0.03** | 0.183 | 0.024 | 0.135 | 0.222 |
|  |  |  |  |  |  |  |  |  |  |

**Supplementary Table 5.** Connectomic characteristics of the 180 ROIs connectivity matrix of the small and large brain group in the male subsample (n = 69). Compare the results with those of Table 3 in the main manuscript. The connectivity ratio was calculated by dividing the interhemispheric by the intrahemispheric connectivity measure. Differences were determined by t-tests for independent samples with 67 degrees of freedom. SD, standard deviation.

Second, a mixed ANOVA model (n = 69) revealed also a significant interaction between brain size in women and connectivity (F_(1,67)_ = 32.6, p = 2.8E-07, η_p_^2^ = 0.33) (Supplementary Fig. 3B). Subsequent post hoc t-tests revealed a significantly and massively increased intrahemispheric connectivity in large compared with small female brains (t_(67)_ = -5.6, p = 4.1E-07, d = 1.34), whereas interhemispheric connectivity was also increased in large compared with small female brains, but with a moderate effect size (t_(67)_ = -3.1, p = 0.003, d = 0.75).

Third, a mixed ANOVA model (n = 69) revealed also a significant interaction between brain size in men and connectivity (F_(1,67)_ = 51.7, p = 6.9E-10, η_p_^2^ = 0.44) (Supplementary Fig. 3C). Subsequent post hoc t-tests revealed a significantly and massively increased intrahemispheric connectivity in large compared with small male brains (t_(67)_ = -6.5, p = 1.0E-08, d = 1.60), whereas interhemispheric connectivity was not significantly different between large and small male brains (t_(67)_ = -2.0, p = 0.047, d = 0.49).

**2.3. Correlation between brain size and type of connectivity**

In addition to these interactional analyses and to further characterize and visualize the relationship between brain size and the types of connectivity, we correlated brain size with the interhemispheric and intrahemispheric connectivity measure. Both correlations were positive and statistically significant, but differed in their strength. The relationship between brain size and intrahemispheric connectivity is strong (r = 0.786, p = 3.6E-30, according Pearson) and brain size explains 62% of the variance in intrahemispheric connectivity. The relationship between brain size and interhemispheric connectivity is heavily reduced in strength (r = 0.384, p = 0.000003) and brain size only explains 15% of the variance in interhemispheric connectivity. The scatter-plots of these relationships are shown in Supplementary Figure 4.


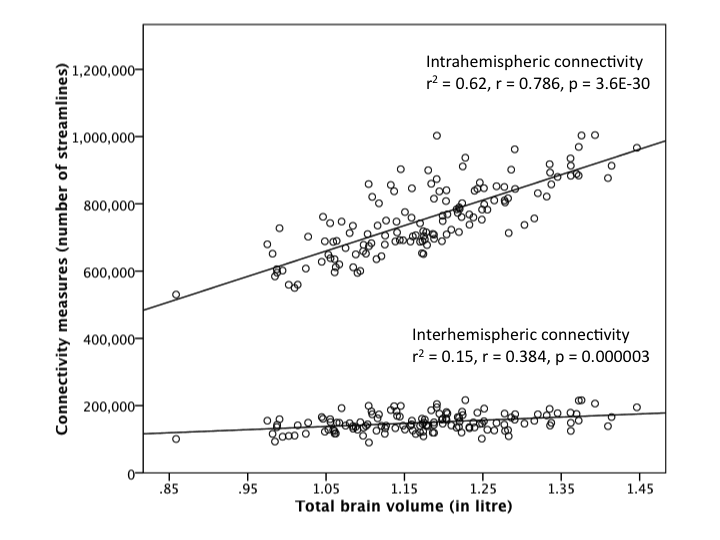


**Supplementary Figure 4.** Correlation between brain size and the interhemispheric as well as intrahemispheric connectivity measure derived from the 180 ROIs connectivity matrix in the gender-pooled sample (N = 138). Although brain size is positively correlated with both connectivity measures, the strengths of these two correlations are quite different. Brain size explains 62% of the variance in intrahemispheric connectivity, whereas brain size explains only 15% of the variance in interhemispheric connectivity.

**2.4. Relationship between brain size and the connectivity ratio**

Beside these mixed ANOVA models and the linear correlations reported above, we also computed the connectivity ratio based on the interhemispheric divided by the intrahemispheric connectivity measure derived from the 180 ROIs connectivity matrix and correlated this ratio with brain size. The mean, standard deviation, minimum, and maximum values as well as the associated error probabilities of the group comparisons of the connectivity ratio derived from the 180 ROIs connectivity matrix can be found in Supplementary Tables 3-5. Across the whole sample (N = 138), the connectivity ratio was significantly negatively correlated with TBV (r = -0.301, p = 0.0003, Pearson correlation). Within the female (n = 69) and male sample (n = 69), however, the connectivity ratios were not statistically significantly correlated with TBV (r = -0.075, p = 0.54 and r = -0.196, p = 0.106, respectively). Plotting the connectivity ratio against TBV (Supplementary Fig. 5), it is obvious that a cubic function better (R^2^ = 0.120, p = 0.0006) explains their relationship than a linear function (R^2^ = 0.091, p = 0.0004), a difference in explained variance that is not merely attributable to the over fitting problem. Please note that without the logarithmical transformation of the TBV values (log_10_ of TBV in liters), the cubic model could not be fitted due to near-collinearity among model terms.


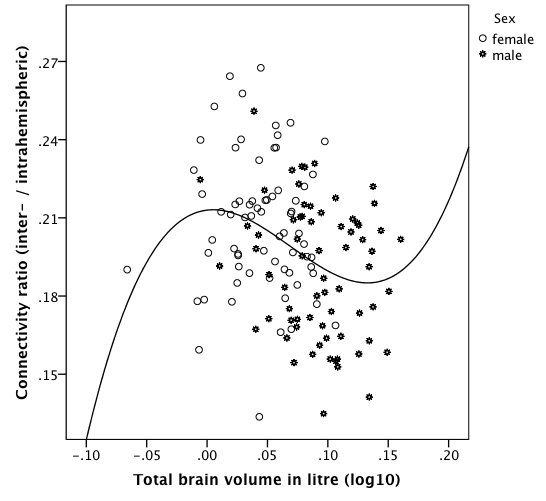


**Supplementary Figure 5.** Relationship between the connectivity ratio derived from the 180 ROIs connectivity matrix and brain size in the gender-pooled sample (N = 138) as revealed by a cubic function. The connectivity ratio was computed based on the number of reconstructed interhemispheric connections (streamlines) divided by the number of intrahemispheric connections. Brains of intermediate size showed the predicted negative association of these measures. However, the smallest (mainly female) and the largest (all male) brains do not follow our predictions. These subjects showed a positive instead a negative association between the connectivity ratio and TBV.

As evidenced in Supplementary Fig. 5, the smallest and the largest brains investigated in the present study showed a positive association between the connectivity ratio and TBV, whereas brains of intermediate size showed the predicted negative association of these measures. We then computed the distribution points of the cubic function (y = 0.054485 * x -5.572829 * x^2^ + 26.949297 * x^3^ + 0.212974) and excluded all subjects below the saddle (log_10_ of TBV in liter = 0.0051 corresponding to 1.012 liter TBV) and above the valley (log_10_ of TBV in liter = 0.1328 corresponding to 1.358 liter TBV) and recomputed the linear correlation of the remaining subjects.

In this way, the 10 smallest brains (9 females) and the 11 largest brains (all males) were excluded. Across the reduced gender-pooled sample (n = 108) the connectivity ratio was negatively correlated with TBV (r = -0.357, p = 0.00004 one-tailed, according Pearson). Within the reduced female sample (n = 60) the connectivity ratio was statistically significantly correlated with TBV (r = -0.245, p = 0.0295 one-tailed), whereas within the reduced male sample (n = 59) the connectivity ratio also correlated negatively with TBV (r = -0.186), but only on a trend level toward statistical significance (p = 0.085 one-tailed).

To sum up, the smallest (female) and the largest (male) brains do not follow our predictions. These subjects showed a positive instead a negative association between the connectivity ratio and TBV. The correlations reported here for the 180 ROIs connectivity matrix are comparable with those derived from the 90 ROIs connectivity matrix (see main manuscript).

**2.5. Cross-species comparison**

The corpus callosum mid-sagittal area, brain size, and the ratio between these two measures are reported in Supplementary Table 6. Figure 5 in the main manuscript shows the scatter plot when regressing the log_10_ of the brain size against the ratio between corpus callosum mid-sagittal area and brain size. The following animal categories were used for the regression; elephants, humans, carnivores, other primates, and rodents. The explained variance of this linear regression is R^2^ = 0.644.

**Supplementary Table 6**

|  |  |  |  |  |  | **Brain size** | **CC** | **Ratio (CC area/** |
| --- | --- | --- | --- | --- | --- | --- | --- | --- |
| **Genus / species** | **Common name** | **N** | **Sex (f/m)** | **Study reported** | **Source study** | **(g or cm3)** | **area (cm2)** | **brain size*100)** |
| **Elephants** |  |  |  |  |  |  |  |  |
| Loxodonta africana | African elephant | 1 | m | Manger et al., 2010 | Manger et al., 2010 | 5145 | 8.52 | 0.166 |
| Loxodonta africana | African elephant | 1 | m | Manger et al., 2010 | Manger et al., 2010 | 5250 | 10.19 | 0.194 |
| Loxodonta africana | African elephant | 1 | m | Manger et al., 2010 | Manger et al., 2010 | 4835 | 9.19 | 0.190 |
| Loxodonta africana | African elephant | 1 | f | Manger et al., 2010 | Hakeem et al., 2005 | 4027 | 12.80 | 0.318 |
| Elephas maximus | Asian elephant | 1 | m | Manger et al., 2010 | Anthony, 1938 | 4460 | 8.09 | 0.181 |
| Elephas maximus | Asian elephant | 1 | f | Manger et al., 2010 | Shoshani et al., 2006 | 5220 | 12.57 | 0.241 |
|  | **sum** | **6** |  |  | **mean** | **4822.8** | **10.23** | **0.215** |
| **Humans*** |  |  |  |  |  |  |  |  |
| Homo sapiens | Human | 69 | 55f/14m | (Small brain group) | Present study | 1089 | 6.66 | 0.612 |
| Homo sapiens | Human | 69 | 14f/55m | (Large brain group) | Present study | 1264 | 7.09 | 0.561 |
| Homo sapiens | Human | 6 | 3f/3m | Rilling & Insel, 1999 | Rilling & Insel, 1999 | 1299 | 6.90 | 0.531 |
| Homo sapiens | Human | 100 | f | Leonard et al., 2008 | Leonard et al., 2008 | 1171 | 5.42 | 0.463 |
| Homo sapiens | Human | 100 | m | Leonard et al., 2008 | Leonard et al., 2008 | 1338 | 5.94 | 0.444 |
| Homo sapiens | Human | 49 | f | Jäncke et al., 1997 | Jäncke et al., 1997 | 1120 | 6.64 | 0.593 |
| Homo sapiens | Human | 71 | m | Jäncke et al., 1997 | Jäncke et al., 1997 | 1240 | 6.63 | 0.535 |
|  | **sum** | **464** |  |  | **mean** | **1217.3** | **6.47** | **0.534** |
| **Carnivores** |  |  |  |  |  |  |  |  |
| Panthera leo | Lion | 1 | n.a. | Manger et al., 2010 | Anthony, 1938 | 231 | 1.16 | 0.502 |
| Hyaena striata | Hyena | 1 | n.a. | Manger et al., 2010 | Anthony, 1938 | 198 | 0.82 | 0.412 |
| Felis caracal | Domestic cat | 1 | n.a. | Manger et al., 2010 | Anthony, 1938 | 54 | 0.43 | 0.787 |
| Ursus arctos | Brown bear | 1 | n.a. | Manger et al., 2010 | Anthony, 1938 | 400 | 2.37 | 0.593 |
| Ursus maritimus | Ice bear | 1 | n.a. | Manger et al., 2010 | Anthony, 1938 | 470 | 2.33 | 0.496 |
| Helarctos malayanus | Sun bear | 1 | n.a. | Manger et al., 2010 | Anthony, 1938 | 217 | 1.16 | 0.532 |
| Canis lupus | Wolf | 1 | n.a. | Manger et al., 2010 | Anthony, 1938 | 130 | 0.98 | 0.750 |
| Canis vulpes | Red fox | 1 | n.a. | Manger et al., 2010 | Anthony, 1938 | 44 | 0.50 | 1.125 |
| Lutra lutra | Otter | 1 | n.a. | Manger et al., 2010 | Anthony, 1938 | 34 | 0.37 | 1.088 |
| Mustela putorius | Ferret | 1 | n.a. | Manger et al., 2010 | Manger et al., 2010 | 8.3 | 0.07 | 0.867 |
|  | **sum** | **10** |  |  | **mean** | **178.6** | **1.02** | **0.715** |
| **Other primates than humans** |  |  |  |  |  |  |  |  |
| Pan paniscus | Bonobo | 1 | n.a. | Manger et al., 2010 | Rilling & Insel, 1999 | 322.4 | 2.73 | 0.848 |
| Pan troglodytes | Chimpanzee | 1 | n.a. | Manger et al., 2010 | Rilling & Insel, 1999 | 349.4 | 2.77 | 0.792 |
| Gorilla gorilla | Gorilla | 1 | n.a. | Manger et al., 2010 | Rilling & Insel, 1999 | 397.3 | 2.96 | 0.745 |
| Pan pygmaeus | Orang utan | 1 | n.a. | Manger et al., 2010 | Rilling & Insel, 1999 | 421.6 | 3.19 | 0.757 |
| Hylobates lar | Gibbon | 1 | n.a. | Manger et al., 2010 | Rilling & Insel, 1999 | 86.0 | 1.07 | 1.245 |
| Papio cynocephalus | Baboon | 1 | n.a. | Manger et al., 2010 | Rilling & Insel, 1999 | 148.5 | 1.24 | 0.837 |
| Macaca mulatta | Macaque | 1 | n.a. | Manger et al., 2010 | Rilling & Insel, 1999 | 82.0 | 1.03 | 1.258 |
| Cercocebus atys | Mangabey | 1 | n.a. | Manger et al., 2010 | Rilling & Insel, 1999 | 102.8 | 1.02 | 0.988 |
| Cebus apella | Capuchin | 1 | n.a. | Manger et al., 2010 | Rilling & Insel, 1999 | 68.9 | 0.79 | 1.144 |
| Saimiri sciureus | Squirrel monkey | 1 | n.a. | Manger et al., 2010 | Rilling & Insel, 1999 | 23.9 | 0.44 | 1.826 |
| Chlorocebus aethiops | Vervet monkey | 1 | n.a. | Manger et al., 2010 | Fears et al., 2009 | 70.3 | 0.89 | 1.266 |
| Galago demidovii | Lesser galago | 1 | n.a. | Manger et al., 2010 | Bauchot & Stephan, 1961 | 3.4 | 0.03 | 0.947 |
| Perodicticus potto | Lory | 1 | n.a. | Manger et al., 2010 | Bauchot & Stephan, 1961 | 14.0 | 0.16 | 1.150 |
| Lemur macaco | Lemur | 1 | n.a. | Manger et al., 2010 | Saban et al., 1990 | 23.6 | 0.21 | 0.890 |
| Daubentonia madagascariensis | Aye-aye | 1 | n.a. | Manger et al., 2010 | Saban et al., 1990 | 45.2 | 0.37 | 0.819 |
| Lagothrix lagothrica | Brown wolley monkey | 1 | n.a. | Manger et al., 2010 | Saban et al., 1990 | 101.0 | 0.39 | 0.386 |
| Papio hamadryas | Baboon | 1 | n.a. | Manger et al., 2010 | Saban et al., 1990 | 201.0 | 1.16 | 0.577 |
|  | **sum** | **17** |  |  | **mean** | **144.8** | **1.20** | **0.969** |
| **Rodents** |  |  |  |  |  |  |  |  |
| Tatera brantsii | Gerbil (mouse) | 1 | n.a. | Manger et al., 2010 | Manger et al., 2010 | 1.61 | 0.03 | 1.677 |
| Thryonomys swinderianus | Greater can rat | 1 | n.a. | Manger et al., 2010 | Manger et al., 2010 | 13.75 | 0.13 | 0.960 |
| Hystrix africaeaustralis | Porcurpine (pic) | 1 | n.a. | Manger et al., 2010 | Manger et al., 2010 | 38.87 | 0.38 | 0.975 |
| Hydrochaeris hydrochaeris | Capybara (pic) | 1 | n.a. | Manger et al., 2010 | Anthony, 1938 | 60 | 0.45 | 0.742 |
|  | **sum** | **4** |  |  | **mean** | **28.6** | **0.25** | **1.088** |

**Supplementary Table 6.** Cross-species comparison of brain size, corpus callosum area, and its ratio. It is obvious that with decreasing brain size (from elephants to humans to carnivores to other primates to rodents) there is an increase in the ratio between the corpus callosum mid-sagittal area and brain size. A non-parametric Kruskal-Wallis test indicated that these ratios are significantly different among elephants, humans, carnivores, other primates, and rodents (n = 44, χ^2^_(4)_ = 25.40, p = 0.00004, φ = 0.76). This is evidence that interhemispheric connectivity scales with negative allometry on brain volume across different mammalian species. *, brain size in humans was measures in cm^3^; CC, corpus callosum; f, female; n, number of species investigated; n.a., not available; m, male.

**3. Supplementary References**

Aboitiz, F., Scheibel, A.B., Fisher, R.S., and Zaidel, E. (1992a). Individual differences in brain asymmetries and fiber composition in the human corpus callosum. *Brain Res.* 598**,** 154-161.

Aboitiz, F., Scheibel, A.B., and Zaidel, E. (1992b). Morphometry of the Sylvian fissure and the corpus callosum, with emphasis on sex differences. *Brain* 115**,** 1521-1541.

Lamantia, A.S., and Rakic, P. (1990a). Axon overproduction and elimination in the corpus callosum of the developing rhesus monkey. *J. Neurosci.* 10**,** 2156-2175.

Lamantia, A.S., and Rakic, P. (1990b). Cytological and quantitative characteristics of four cerebral commissures in the rhesus monkey. *J. Comp. Neurol.* 291**,** 520-537.

Nowicka, A., and Tacikowski, P. (2011). Transcallosal transfer of information and functional asymmetry of the human brain. *Laterality* 16**,** 35-74.

Ringo, J. (1991). Neuronal Interconnection as a Function of Brain Size. *Brain Behav. Evol.* 38**,** 1-6.

Ringo, J., Doty, R.W., Demeter, S., and Simard, P.Y. (1994). Time Is of the Essence: A Conjecture that Hemispheric Specialization Arises from Interhemispheric Conduction Delay. *Cereb. Cortex* 4**,** 331-343.

Van Der Knaap, L.J., and Van Der Ham, I.J. (2011). How does the corpus callosum mediate interhemispheric transfer? A review. *Behav. Brain Res.* 223**,** 211-221.
